# Patient Adoption of Digital Use Cases in Family Medicine and a Nuanced Implementation Approach for Family Doctors: Quantitative Web-Based Survey Study

**DOI:** 10.2196/58867

**Published:** 2025-03-05

**Authors:** Julian Beerbaum, Sibylle Robens, Leonard Fehring, Achim Mortsiefer, Sven Meister

**Affiliations:** 1 Health Care Informatics, Faculty of Health School of Medicine Witten/Herdecke University Witten Germany; 2 Department of Psychology and Psychotherapy Witten/Herdecke University Witten Germany; 3 Department of Gastroenterology Helios University Hospital Wuppertal Witten/Herdecke University Wuppertal Germany; 4 Faculty of Health School of Medicine Witten/Herdecke University Witten Germany; 5 General Practice II and Patient-Centeredness in Primary Care, Institute of General Practice and Primary Care Faculty of Health, School of Medicine Witten/Herdecke University Witten Germany; 6 Department Healthcare Fraunhofer Institute for Software and Systems Engineering Dortmund Germany

**Keywords:** technology acceptance, UTAUT, family doctor, digital health, eHealth, video consultation, electronic health records, digital anamnesis, online appointment scheduling

## Abstract

**Background:**

Digital use cases describe the application of technology to achieve specific outcomes. Several studies in health care have examined patients’ overall attitudes toward digitalization and specific use cases. However, these studies have failed to provide a comparison of patient acceptance criteria between inherently different digital use cases in family medicine.

**Objective:**

To address this research gap, this paper aimed to assist family doctors in selecting digital use cases by comparing the underlying patient adoption factors and in driving usage of these use cases by presenting a differentiated implementation approach.

**Methods:**

Adapting an established Unified Theory of Acceptance and Use of Technology (UTAUT) questionnaire to 4 digital use cases in family medicine, we surveyed a large cross-sectional sample of adults living in Germany. The results of the web-based survey were then analyzed via descriptive statistics, ANOVA, and hierarchical regression models to compare the effects of sociodemographic and technology acceptance factors on the intention to use a specific use case.

**Results:**

Our web-based survey included 1880 participants. Of these 1880 participants, only 304 (16.2%) agreed that the degree of digitalization is important when selecting a family practice. However, more digitally literate participants attributed greater importance to this criterion (B=0.226, SE 0.023; β=.223; *P*<.001), and digital literacy was found to be dependent on age (Welch *F*_3,968.29_=53.441; *P<*.001). Regarding sociodemographic characteristics, only digital literacy demonstrated a significant effect on the intention to use for all use cases, particularly scheduling doctor appointments online (B=0.322, SE 0.033; β=.408; *P<*.001). Furthermore, *performance expectancy* was the strongest predictor of the intention to use for all use cases, while further effects of technology acceptance factors depended on the use case (receiving medical consultations via video: B=0.603, SE 0.049; β=.527; *P<*.001; scheduling doctor appointments online: B=0.566, SE 0.043; β=.513; *P<*.001; storing personal medical information via electronic health records: B=0.405, SE 0.047; β=.348; *P<*.001; and providing personal information before consultation digitally [digital anamnesis]: B=0.434, SE 0.048; β=.410; *P<*.001). To illustrate, *perceived privacy and security* had an effect on the intention to use electronic health records (B=0.284, SE 0.040; β=.243; *P<*.001) but no effect on the intention to use video consultations (B=0.068, SE 0.042; β=.053; *P=*.10).

**Conclusions:**

In the selection and implementation of digital use cases, family doctors should always prioritize the perceived value of the digital use case for the patient, and further criteria might depend on the digital use case. Practice owners should therefore always harmonize the introduction of digital use cases with their own patient care strategies. Not every digital innovation fits every strategy and therefore every practice.

## Introduction

### Background

The benefits of applying digital use cases, which we understand as the application of digital technologies to achieve a specific outcome, are well documented for the health care sector [1,2]. For example, in family medicine in Germany, where the family doctor is the first contact point to the health system and thus particularly important to ensure adequate treatment [3], digital use cases could address overarching challenges such as the shortage of family doctors [4]. After years of sporadic offering and use of digital tools in family medicine, COVID-19 accelerated their use [2,5]. Furthermore, this trend toward digitalization increased the diversity of digitally enabled use cases (eg, from online appointment systems to digital anamnesis tools and video consultation offerings) [5]. To illustrate this, the American Medical Association showed in 2022 in their longitudinal survey that the average number of digital applications used by a doctor increased by 72% between 2016 and 2022 [6].

It is apparent that, today more than ever, family doctors can decide which of the various offerings to implement. However, overall adoption rates of digital health care use cases by both patients and family doctors are still at low levels in some regions. For example, in Germany in 2023, only about 1% of all statutorily insured patients stored personal medical information via electronic health records [7,8]. A similar picture has emerged in other countries (eg, only about 3% of medical appointments in November 2023 in general practice in the United Kingdom were conducted via video) [9]. To stimulate broader adoption of digital use cases, legislative bodies in countries, such as the United Kingdom and Germany, revised their regulatory frameworks, such as directing the default use of electronic health records in Germany [10,11]. Nevertheless, to translate these incentives into actual patient usage of digital use cases, we argue that it is vital to assess the underlying factors determining patient adoption behavior. This evaluation is particularly important as family doctors consider patient expectations when deciding to offer patient-facing use cases [12,13].

In assessing technology acceptance, we premise that differentiation and comparison between use cases might offer fruitful insights, as we presume inherent structural differences between digital use cases (eg, degree of voluntariness of use for patients or degree of dealing with sensitive medical information). Thereby, we add to the academic discourse since there are several studies in health care examining patients’ overall digital attitudes toward digitalization [14] as well as for specific use cases [15-18]. However, these studies have failed to provide a nuanced assessment and comparison of patient acceptance criteria between distinct digital use cases in family medicine. This differentiation is particularly vital as potentially diverse patient technology acceptance criteria per use case might emerge, and therefore, a differentiated approach to drive adoption might be needed.

### Objectives

This survey-based study provides a nuanced comparison of patients’ technology acceptance of key digital use cases in family medicine (ie, receiving medical consultations via video, scheduling doctor appointments online, storing personal medical information via electronic health records, and providing personal information before consultation digitally [digital anamnesis]).

Accordingly, this paper aims to support family doctors in selecting digital use cases by showcasing their relative importance to patients and in driving their use. In doing so, it highlights the need for a differentiated implementation approach based on different patient acceptance criteria for each digital use case.

For the scope of this research paper, we focused on family doctors as they act as gatekeepers for access to digital technologies [19]. Additionally, we hypothesized that family doctors could be important for driving adoption with an already established and trusted patient-doctor relationship.

## Methods

### Research Approach

Building on the insights of a rapid literature review on patient acceptance of 4 digital use cases, we leveraged an existing questionnaire assessing the acceptance of electronic health records in family medicine based on the Unified Theory of Acceptance and Use of Technology (UTAUT) model [20] and adapted it to the other use cases. The objective of our convenience sample approach was to gather a cross-sectional sample of adult patients living in Germany.

### Digital Use Cases in Family Medicine

Family medicine typically provides the first medical contact within the health care system [21], and substantial evidence supports the application of digital technologies (digital use cases) in this setting [1,2]. Due to the high pace of innovation and great variety of digital use cases in family medicine, we limited the number of use cases in scope. The final selection of digital use cases was a joint decision of a multi-professional team (comprising 4 researchers with diverse backgrounds in the digitalization of family medicine and 1 practicing family doctor). The decision was based on (1) the applicability of these use cases for patients in family medicine and (2) their current perceived practical relevance for both patients and family doctors. Thereby, we adhered to the following use case definitions:

- Receiving medical consultations via video: We followed the definition by Keuper et al [22] that “a video consultation is considered as a real-time visual and audio (digital) contact moment between the patient and health care provider.” This excludes, for example, telehealth applications that do not facilitate a contact moment (eg, telemonitoring [23]).
- Scheduling doctor appointments online: We understand online appointment scheduling as the use case of booking an appointment via the internet in real-time [24].
- Storing personal medical information via electronic health records: We followed the definition of the National Coordinator for Health Information Technology of the United States that “EHRs are real-time, patient-centered records that make information available instantly and securely to authorized users” [25].
- Providing personal information before consultation digitally (digital anamnesis): Patient anamnesis refers to the data that patients provide about their personal medical history [26]. Digital anamnesis then applies this concept to the digital space as the information-gathering process occurs via a digital application.

Nevertheless, the full scale of adoption of the various digital use cases is yet to be realized [27]. Overall barriers can be pinpointed to a fragmentation of the digital health landscape, inadequate legislative frameworks, and limited clarity on information exchange, highlighting the necessity to consider the patients’ perspectives even more [28].

### Research Model

Our research attempts to compare different use cases in family medicine and highlight substantial differences between factors in patient technology acceptance.

Technology acceptance describes the positive decision of an individual toward using technology and explains the various factors and contexts incentivizing individuals to use a particular technology [29,30]. This range of factors in the decision-making process to use a tool in a health care setting is reflected in the various theories used to describe this technology acceptance. AlQudah et al [30] and Heinsch et al [31] demonstrated that the Technology Acceptance Model (TAM) and the UTAUT are the most applied theories in health care settings. Nevertheless, many studies combine different theories to explain technology acceptance with various factors [32,33].

The original UTAUT model consists of 4 constructs. The definitions of these UTAUT constructs by Venkatesh et al [34] are as follows. *Performance expectancy* is defined as “the degree to which an individual believes that using the system will help him or her to attain gains in job performance.” In the family medicine setting, this translates to the usefulness of the use case to manage one’s health or disease in a family practice setting. *Effort expectancy* is defined as “the degree of ease associated with the use of the system” [34], which, in our setting, relates to how easily patients can use the digital use case. *Social influence* is defined as “the degree to which an individual perceives that important others believe he or she should use the new system” [34]. In family medicine, these important others could range from physicians to public opinions to friends and family members. *Facilitating condition* is defined as “the degree to which an individual believes that an organizational and technical infrastructure exists to support use of the system” [34], which, in our context, is associated with having the right resources, compatible technologies, and knowledge to use the digital use case. The additionally included dimension in our study *perceived privacy and security* refers to “the degree to which patients believe that the respective use case is safe from intrusion and personal information is protected” [20,35]. We support the justification of Abd-Alrazaq et al [20] for applying the UTAUT model in the family medicine setting as the model has shown explanatory power in comparable contexts [31].

In line with our objective to assist family doctors by demonstrating the relative importance of digital use cases for patients and by highlighting different patient adoption factors, we tested 3 key hypotheses (hypothesis A, hypothesis B, and hypothesis C).

First, to illustrate the relative importance of each use case for patients, we hypothesized that awareness, availability, and actual usage behavior differ across use cases. For example, commercial studies have shown that 78% of surveyed patients wish for online appointment scheduling at their family doctor, while only 32% wish for an electronic health record and 27% wish for a video consultation [36].

Hypothesis A is as follows: Different use cases have differences in awareness, availability, and actual usage by patients.

Second, to determine the underlying adoption factors of digital use cases in family medicine, we formulated hypotheses B and C. Our rapid literature review, which aligned with the Preferred Reporting Items for Systematic Reviews and Meta-Analyses (PRISMA) extension for Rapid Reviews (PRISMA-RR) and recommendations by Tricco et al [37] and King et al [38], showed that different use cases have different factors driving technology acceptance [39,40]. The detailed approach and results can be found in the checklist in Multimedia Appendix 1.

Hypothesis B is as follows: Different use cases have different significant technology acceptance factors driving behavioral intention to use.

Various studies have indicated that sociodemographic factors affect technology acceptance. Mueller et al [4] argued that living in rural areas with potentially less doctor density might increase the likelihood of using digital offerings such as video consultation as it “saves the trip to the physician’s practice.” Thus, we hypothesized that the degree of the intended use of digital offerings varies (eg, by region and size of a city).

Hypothesis C is as follows: Different use cases have different sociodemographic characteristics impacting behavioral intention to use.

**Figure 1:**
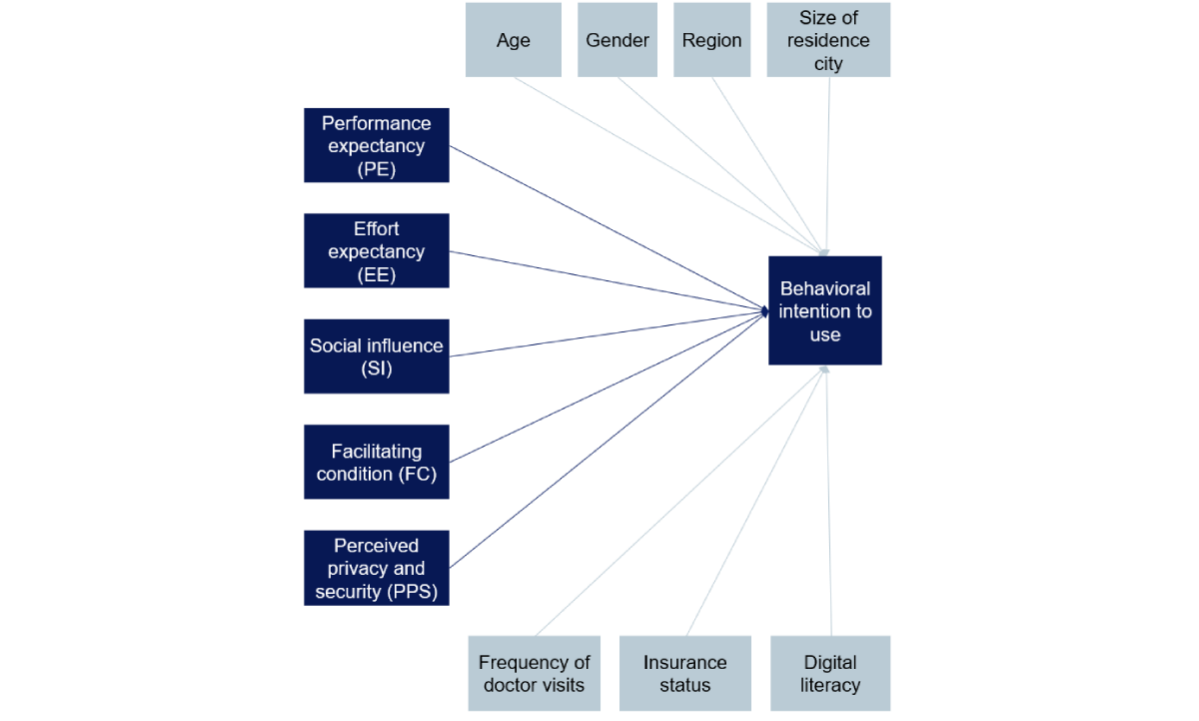
Research model. The effects of sociodemographic and technology acceptance factors on patients’ intention to use a digital use case in family medicine in Germany in 2023 [20]. Dark blue indicates areas to collect evidence for hypothesis B, while gray indicates areas to test hypothesis C.

While hypothesis A pertains to indicated usage behavior, hypotheses B and C test the effect of a respective technology acceptance factor on the behavioral intention to use a specific digital use case in family medicine. The research model is depicted in Figure 1.

In these superordinate hypotheses, we replicated the same research model for each digital use case, with each testing the same subordinate hypotheses. Therefore, all subordinate hypotheses have the same components.

- Component 1 (illustrated in the research model): Respective technology acceptance factor [B]/respective sociodemographic characteristic [C] positively influences behavioral intention to use …
- Component 2: … a respective digital use case [online-booking platforms; digital anamnesis tools; video consultation platforms; electronic health records]

### Survey Construction and Design

The questionnaire was constructed based on a rapid literature review on technology acceptance of digital use cases in family medicine (Multimedia Appendix 1) and our research model. Given its demonstrated validity and reliability in a family medicine setting (eg, average Cronbach α of .95; average “average variance contracted” [AVE] of 0.95; and average composite reliability [CR] value of 0.87), we leveraged an existing questionnaire assessing a comparable use case in a family medicine setting in the United Kingdom [20]. The questionnaire extends the UTAUT model [34] with additional items on *perceived privacy and security* [35], which also emerged in our literature review as an additional element to understand technology acceptance in health care settings [18,40]. Dropped items with lower factor loadings in the original model of Abd-Alrazaq et al [20] were excluded from our questionnaire. For data restriction purposes, our study did not look at the actual use behavior.

To ensure construct comparability in different use cases, we replaced “patient portal” in the original questionnaire with the names of the other use cases. The questionnaire was then translated into German based on existing validated translations of the UTAUT construct [41] and pretested with 21 participants from the sample target group. Minor adjustments to answer options, the order of questions, and the wording of selected items were made (the translated survey can be found in Multimedia Appendix 2). From a methodological viewpoint, the survey study was aligned with CHERRIES (Checklist for Reporting Results of Internet E-Surveys) (Multimedia Appendix 3). The web-based survey was finally conducted between May 30, 2023, and November 22, 2023, using the software LimeSurvey (LimeSurvey GmbH).

Participants were shown brief descriptions of all the digital use cases in the scope of this paper to minimize any misunderstanding. Participants were then randomly assigned to 2 of the digital use cases for which they answered the technology acceptance items on a Likert scale. The number of use cases per participant was later reduced from 2 to 1 due to higher-than-expected dropout rates. This adjustment was appropriate as the order of questions was not changed.

### Recruitment and Sample Composition

Our convenience sampling approach aimed to recruit a large cross-sectional sample of adults living in Germany. Since the only limiting factor to participate in the study was reaching legal age, our sample was broad, which is reflected in the variety of recruitment channels. Participants were mainly recruited via the Social Sciences’ Panel, a convenience pool project for scientific research, as well as social media platforms such as LinkedIn, Instagram, and Facebook; online (research) forums; and flyers in the waiting rooms of family practices and other facilities. The questionnaire was only posted online in the open-access mode, and no responses from other sources (eg, physical questionnaires) were manually added. Further details, such as the wording of the advertisement of the survey, can be found in Multimedia Appendix 3.

Applied data cleaning mechanisms included removing duplicate entries, excluding the fastest 5% responses, eliminating entries with no reasonable answer behavior (straight lining or incorrectly answering 2 control questions), and deleting entries with no specified demographics and those not meeting the k≥5 criteria [42,43]. We believe that these data-cleaning measures contributed to the validity and reliability of our study. First, we avoided overrepresentation of multiple entries by single participants. Second, we enhanced response quality by minimizing obvious nondiligent answer behavior. Finally, we ensured meaningful analysis by eliminating “not specified” responses while at the same time adhering to our privacy policy by deleting responses that failed the k≥5 criteria.

**Figure 2:**
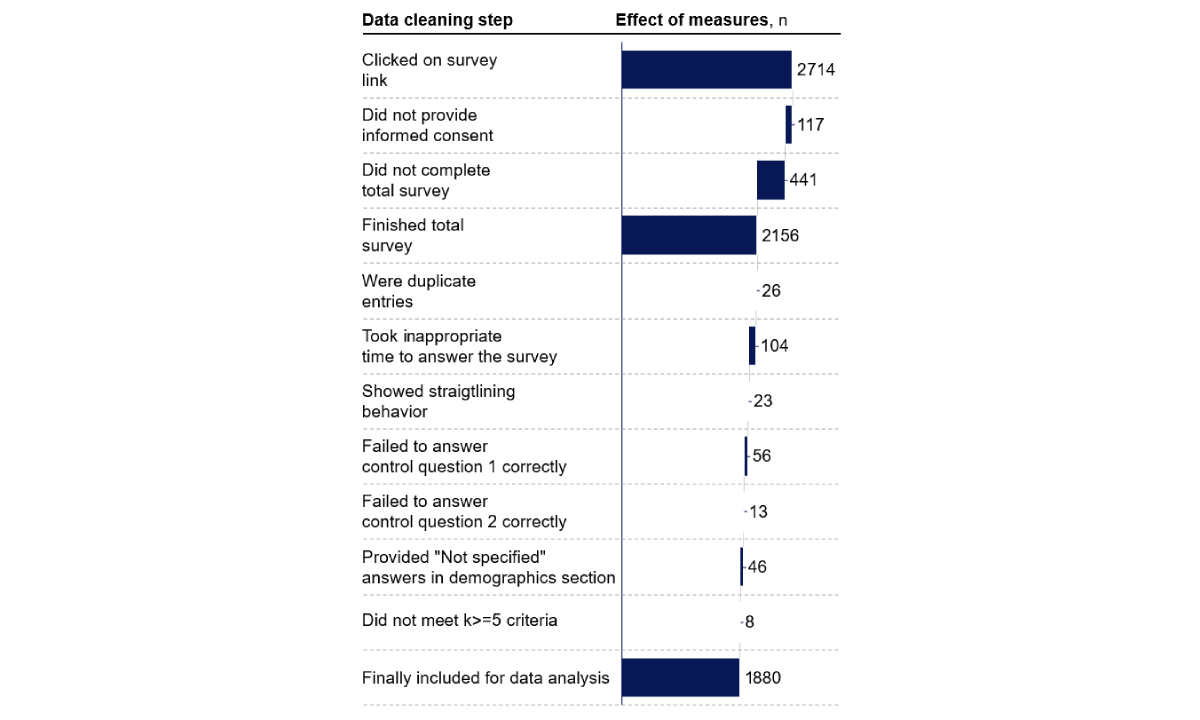
Included responses for data analysis. Impact of dropout and data cleaning measures.

Details of data cleaning measures can be found in Figure 2.

### Empirical Analysis

Validation of the research model and statistical analysis via IBM SPSS 29 were performed separately for each use case in 3 steps.

First, Cronbach α values were calculated to evaluate the internal consistency of the study. The acceptable cutoff point in line with our exploratory research design was set at .60 [44]. Second, descriptive statistics were applied to test hypothesis A that different use cases have differences in awareness, availability, and actual usage among patients. Third, to test our hypotheses B and C regarding whether different use cases have different significant factors influencing behavioral intention to use, each research model was tested separately via multiple hierarchical regression. Thus, we tested the subordinated hypotheses for each digital use case.

The hierarchical regression was adequate to differentiate between the effects of technology acceptance and sociodemographic factors on intention to use. Therefore, we collected evidence for hypotheses B and C with a consistent analytical approach. Additionally, given the 4 different research models and a subsequent high number of subordinate hypotheses, a robust yet straightforward analytical approach was deemed necessary. The statistical significance level was set at *P<*.05 [45].

The first block entered into each model included only sociodemographic variables (age, gender, region of residency, size of the residence city, frequency of doctor visits, insurance status, and digital literacy). This allowed us to isolate the effects of different sociodemographic factors on intention to use, validating hypothesis C. The second block added the adapted UTAUT factors into the model (*performance expectancy*, *effort expectancy*, *social influence*, *facilitating condition*, and *perceived privacy and security*). The final model addressed hypothesis B, testing the effect of technology acceptance factors on the intention to use a specific use case.

Four requirements for multiple regression models have been tested and confirmed for each of the use cases. First, multicollinearity can be rejected as all variance inflation factor (VIF) values were below 10 [46]. Second, autocorrelation can be rejected as the Durbin-Watson value for each use case was close to 2 [47]. Third, the normal distribution of residuals can be confirmed after visual inspection of the histogram of the standardized residuals. Lastly, homoscedasticity can be assumed as the regression of the standardized residual versus standardized predicted value scatter plot did not yield any considerable pattern.

By assessing usage rates, testing the technology acceptance model for each digital use case, and comparing our results across use cases, we collected evidence to assess our hypotheses A, B, and C. We accepted or rejected these superordinated hypotheses based on varying usage rates or different factors impacting the behavioral intention to use in different use cases (the decision was further validated with our research team).

### Ethical Considerations

Our human subject research study was approved by the Ethics Committee of Witten/Herdecke University (Nr. S-245/2022). All participants provided informed consent in line with the GDPR (General Data Protection Regulation) guidelines prior to the start of the survey and had the ability to opt-out. No personal data were gathered, and sociodemographic data points were anonymized in accordance with k≥5 anonymity. All participants filled out the questionnaire voluntarily, and no compensation was offered.

## Results

### Internal Consistency and Sample Size

We calculated Cronbach α values for all 5 technology acceptance factors and the intention to use a specific digital use case. Therefore, we validated 24 variables across all 4 models. Of the 24 variables across all use cases, 23 met the threshold of >.60 [48], while 1 variable showed a Cronbach α of .57 (scheduling doctor appointments online: facilitating condition). The Cronbach α values for all 5 technology acceptance factors and intention to use are shown in Table 1.

Additionally, we leveraged the convenience sampling strategy to gather a large cross-sectional sample of German-speaking adults. Our final sample included 1880 adult respondents living in Germany. The demographic characteristics of the sample are shown in Table 2.

As the number of assigned use case assessments was reduced during the study, 175 out of the 1880 participants initially assessed the technology acceptance of 2 digital use cases, resulting in a total of 2055 technology acceptance assessments, with limited overlap between the groups (receiving medical consultations via video: n=494 [number of respondents who also assessed other use cases: n=80]; scheduling doctor appointments online: n=542 [number of respondents who also assessed other use cases: n=75]; storing personal medical information via electronic health records: n=528 [number of respondents who also assessed other use cases: n=103]; providing personal information before consultation digitally [digital anamnesis]: n=491 [number of respondents who also assessed other use cases: n=92]).

**Table 1:** Construct reliability (Cronbach α) in each digital use case.

Variable	Number of items	Digital use case			
		Receiving medical consultations via video	Scheduling doctor appointments online	Storing personal medical information via electronic health records	Providing personal information before consultation digitally (digital anamnesis)
Behavioral intention to use	3	.91	.90	.92	.90
Performance expectancy	3	.88	.85	.85	.87
Effort expectancy	4	.75	.71	.74	.76
Social influence	3	.91	.92	.91	.92
Facilitating condition	3	.70	.57	.60	.62
Perceived privacy and security	3	.71	.61	.74	.73

**Table 2:** Characteristics of the adult respondents living in Germany in 2023.

Variable		Value (N=1880)
**Gender, n (%)**		
	Male	821 (43.7)
	Female	1059 (56.3)
**Age group (years), n (%)**		
	18-30 years	295 (15.7)
	31-50 years	519 (27.6)
	51-65 years	608 (32.3)
	>65 years	458 (24.4)
**Frequency group (visits in the last 12 months), n (%)**		
	0	132 (7.0)
	1	362 (19.3)
	2-3	705 (37.5)
	>3	681 (36.2)
**Insurance status, n (%)**		
	Statutorily	1459 (77.6)
	Privately	421 (22.4)
**Population size of residence (inhabitants), n (%)**		
	≤20,000	525 (27.9)
	20,001-200,000	581 (30.9)
	>200,000	774 (41.2)
**Region, n (%)**		
	East	437 (23.2)
	North	326 (17.3)
	South	365 (19.4)
	West	752 (40.0)
**Self-assessed digital literacy, n (%)**		
	Low (1-4)	103 (5.5)
	Medium (5-7)	455 (24.2)
	High (8-10)	1322 (70.3)
Digital literacy score, mean (SD)		8.0 (1.8)

### Current Patient Adoption Rates of Digital Use Cases in Family Medicine

There was support for hypothesis A that different use cases have differences in awareness, availability, and actual usage by patients.

Overall, low offering and usage rates of digital use cases were evident. For example, nearly all respondents (1750/1880, 93.1%) were aware of online booking platforms; however, only approximately one-third (617/1880, 32.8%) stated that this service is offered in their family practice. Nevertheless, the vast majority (537/617, 87.0%) of those respondents who were offered online booking services in their family practice reported using them. This usage-to-availability ratio was higher for scheduling doctor appointments online than for video consultations where only less than half of the participants (94/225, 41.8%) stated usage. The results are presented in Table 3. The share of respondents using digital use cases when offered at their family doctor was as follows: receiving medical consultations via video, 94/225 (41.8%); scheduling doctor appointments online, 537/617 (87.0%); storing personal medical information via electronic health records, 108/166 (65.1%); providing personal information before consultation digitally (digital anamnesis), 53/78 (68.0%).

**Table 3:** Patient awareness, availability, and patient usage of selected digital use cases in family medicine in Germany in 2023.

Statement and digital use case		Response option, n (%)				
		Yes	No	I don’t know	Not offered in my family practice	Not specified
**“I am aware of this use case”**						
	VC^a^	1302 (69.3)	571 (30.4)	—^b^	—	7 (0.4)
	OS^c^	1750 (93.1)	130 (6.9)	—	—	0 (0.0)
	EHR^d^	1220 (64.9)	638 (33.9)	—	—	22 (1.2)
	DA^e^	784 (41.7)	1075 (57.2)	—	—	21 (1.1)
**“The use case is offered at my family doctors** **”**						
	VC	225 (12.0)	1092 (58.1)	—	559 (29.7)	4 (0.2)
	OS	617 (32.8)	1031 (54.8)	—	229 (12.2)	3 (0.2)
	EHR	166 (8.8)	690 (36.7)	—	1016 (54.0)	8 (0.5)
	DA	78 (4.0)	956 (50.9)	—	835 (44.4)	11 (1.0)
**“I use this digital use case at my family doctors”**						
	VC	94 (5.0)	909 (48.4)	—	793 (42.2)	84 (5.0)
	OS	537 (28.6)	583 (31.0)	—	712 (37.9)	48 (3.0)
	EHR	108 (5.7)	907 (48.2)	—	622 (33.1)	243 (12.9)
	DA	53 (3.0)	877 (46.6)	—	759 (40.4)	191 (10.2)

^a^VC: receiving medical consultations via video.

^b^Not applicable.

^c^OS: scheduling doctor appointments online.

^d^EHR: storing personal medical information via electronic health records.

^e^DA: providing personal information before consultation digitally (digital anamnesis).

There was a low usage rate of digital use cases as only 16.2% (304/1880) of respondents agreed or fully agreed that the degree of digitalization is key when selecting a family practice. Furthermore, the willingness to change the family practice when a particular digital use case is unavailable was found to depend on the use case (69/1880, 3.7% for receiving medical consultations via video; 200/1880, 10.6% for scheduling doctor appointments online; 131/1880, 7.0% for storing personal medical information via electronic medical records; and 82/1880, 4.4% for providing personal information before consultation digitally [digital anamnesis]). Nevertheless, regression analysis showed that when selecting a family practice, participants with higher digital literacy tended to assign a higher value to the degree of digitalization (B=0.226, SE 0.023; β=.223; *P<*.001).

### Factors Explaining Technology Acceptance in Family Medicine

To better understand the predictors of technology acceptance, we conducted a multiple hierarchical linear regression analysis on the intention to use each use case. Block 1 collects evidence to validate hypothesis C, which involves testing the sole effect of sociodemographic variables on intention to use, while block 2 refers to hypothesis B, which involves validating the effect of technology acceptance factors. In doing so, we did not moderate prior experience with a particular use case to reduce the complexity of each model. The results are illustrated in Table 4. All details of the respective models can be found in Multimedia Appendix 4.

**Table 4:** Standardized coefficients for hierarchical linear regression models explaining patients’ intention to use a specific digital use case in family medicine in Germany in 2023.

Block and variable				Receiving medical consultations via video (n=494)				Scheduling doctor appointments online (n=542)				Storing personal medical information via electronic health records (n=528)				Providing personal information before consultation digitally (digital anamnesis) (n=491)		
				β		*P* value		β		*P* value		β		*P* value		β		*P* value
**Block 1: Sociodemographic variables**																		
	**Frequency of doctor visits (vs no doctor visit in the last 12 months)**																	
		1 visit	.032		.69		.213		.01^a^		–.008		.92		.025		.74	
		2-3 visits	.134		.15		.233		.01^a^		.142		.10		.089		.27	
		>3 visits	.137		.14		.224		.01^a^		.066		.45		.136		.10	
	**Gender (vs male)**																	
		Female	.900		.04^a^		.084		.04^a^		–.101		.02^a^		–.037		.41	
	**Age (vs 18-30 years)**																	
		31-50 years	.087		.13		–.011		.84		–.017		.75		.013		.82	
		51-65 years	.107		.09		–.023		.69		–.021		.71		–.125		.04^a^	
		>65 years	.032		.60		–.083		.16		.020		.74		–.158		.01^a^	
	**Size of the residence city (vs ≤20,000)**																	
		20,001-200,000	–.025		.62		.022		.66		.056		.29		.007		.90	
		>200,000	–.057		.30		.032		.53		.023		.67		–.005		.92	
	**Region (vs East)**																	
		South	–.061		.22		.005		.93		–.028		.57		–.038		.48	
		West	–.061		.25		–.035		.48		–.031		.53		.022		.69	
		North	–.026		.64		.029		.58		.052		.33		.020		.74	
	Type of insurance		–.044		.32		–.010		.81		–.022		.60		.067		.13	
	Digital literacy		.335		<.001^a^		.408		<.001^a^		.329		<.001^a^		.250		<.001^a^	
**Block 2^b^: Sociodemographic variables + technology acceptance factors**																		
	Performance expectancy		.527		<.001^a^		.513		<.001^a^		.348		<.001^a^		.410		<.001^a^	
	Effort expectancy		.135		.005^a^		.218		<.001^a^		.096		.03^a^		.236		<.001^a^	
	Social influence		.206		<.001^a^		.054		.07		.101		<.001^a^		.173		<.001^a^	
	Facilitating condition		.039		.36		.028		.44		.198		<.001^a^		.085		.045^a^	
	Perceived privacy and security		.053		.10		.094		<.001^a^		.243		<.001^a^		.104		.003^a^	

^a^Statistically significant (*P*<.05).

^b^The effect of sociodemographic variables is not displayed as the focus of the analysis is on the effect of technology acceptance factors on intention to use.

All final models (including steps 1 and 2) significantly predicted technology acceptance, whereas the addition of the UTAUT variables in the second step increased the explained variance significantly in all models for all use cases. In the first step, sociodemographic variables alone explained on average 14.6% of the intention to use a specific digital use case (receiving medical consultations via video: *R*^2^=0.124; *F*_14,479_=4.849; *P*<.001; scheduling doctor appointments online: *R*^2^=0.189; *F*_14,527_=8.780; *P*<.001; storing personal medical information via electronic health records: *R*^2^=0.150; *F*_14,513_=6.484; *P*<.001; providing personal information before consultation digitally [digital anamnesis]: *R*^2^=0.121; *F*_14,476_=4.698; *P*<.001). The final models explained on average 62.8% of the variance in intention to use (receiving medical consultations via video: *R*^2^=0.624; *F*_19,474_=41.356; *P<*.001; scheduling doctor appointments online: *R*^2^=0.624; *F*_19,522_=45.617; *P<*.001; storing personal medical information via electronic health records: *R*^2^*=*0.631; *F*_19,508_=45.655; *P<*.001; providing personal information before consultation digitally [digital anamnesis]: *R*^2^=0.631; *F*_19,471_=42.364; *P<*.001).

There was support for hypothesis B that different use cases have different significant technology acceptance factors. Only *performance expectancy* and *effort expectancy* were significant predictors of technology acceptance for all use cases. Additionally, the magnitude of the effects of technology acceptance factors differed between use cases (*performance expectancy*: receiving medical consultations via video: B=0.603, SE 0.049; β=.527; *P<*.001; scheduling doctor appointments online: B=0.566, SE 0.043; β=.513; *P<*.001; storing personal medical information via electronic health records: B=0.405, SE 0.047; β=.348; *P<*.001; providing personal information before consultation digitally [digital anamnesis]: B=0.434, SE 0.048; β=.410; *P<*.001; *effort expectancy*: receiving medical consultations via video: B=0.236, SE 0.084; β*=*.135; *P=*.01; scheduling doctor appointments online: B=0.391, SE 0.075; β=.218; *P<*.001; storing personal medical information via electronic health records: B=0.153, SE 0.070; β*=*.096; *P=*.03; providing personal information before consultation digitally [digital anamnesis]: B=0.366, SE 0.073; β=.236; *P*<.001).

**Figure 3:**
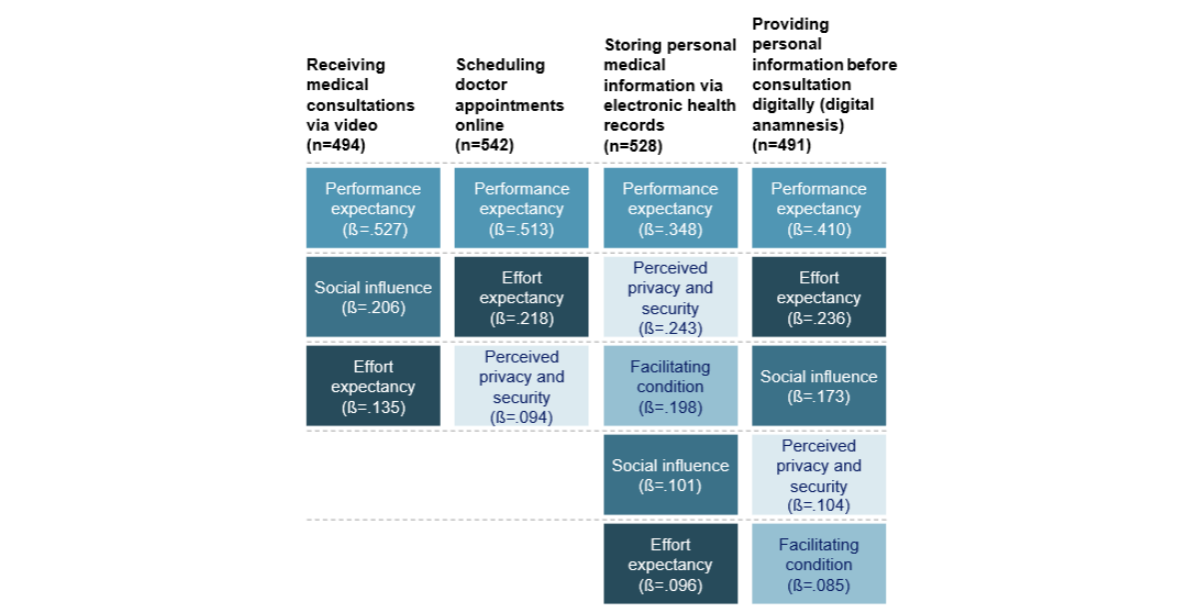
Magnitudes of the effects of technology acceptance factors on the behavioral intention to use a digital use case in family medicine in Germany in 2023 (ranking based on β).

We found that *social influence* was significant for all use cases, except for scheduling doctor appointments online (receiving medical consultations via video: B=0.227, SE 0.036; β=.206; *P<*.001; storing personal medical information via electronic health records: B=0.108, SE 0.033; β*=*.101; *P=*.001; providing personal information before consultation digitally [digital anamnesis]: B=0.186, SE 0.036; β=.173; *P<*.001). Moreover, *perceived privacy and security* was significant for all use cases, except for receiving medical consultations via video (scheduling doctor appointments online: B=0.117, SE 0.037; β=.094; *P=*.002; storing personal medical information via electronic health records: B=0.284, SE 0.040; β=.243; *P<*.001; providing personal information before consultation digitally [digital anamnesis]: B=0.121, SE 0.040; β=.104; *P=*.003). *Facilitating condition* was only significant for storing personal medical information via electronic health records (B=0.299, SE 0.061; β=.198; *P<*.001) and providing personal information before consultation digitally (digital anamnesis) (B=0.127, SE 0.063; β=.085; *P*=.045). A simplified illustration of the different magnitudes of effects is presented in Figure 3.

There was partial support for hypothesis C that different use cases have different sociodemographic factors impacting the behavioral intention, as self-assessed digital literacy had a positive effect for all use cases (receiving medical consultations via video: B=0.341, SE 0.049; β=.335; *P<*.001; scheduling doctor appointments online: B=0.322, SE 0.033; β=.408; *P<*.001; storing personal medical information via electronic health records: B=0.308, SE 0.041; β=.329; *P<*.001; providing personal information before consultation digitally [digital anamnesis]: B=0.239, SE 0.044; β=.250; *P<*.001).

Further, significant effects of sociodemographic variables on intention to use depended on the use case. Gender (female vs male) had an effect on 3 digital use cases, but the direction of that effect varied (receiving medical consultations via video: B=0.321, SE 0.159; β=.090; *P=*.04; scheduling doctor appointments online: B=0.247, SE 0.122; β=.084; *P=*.04; storing personal medical information via electronic health records: B=–0.356, SE 0.149; β=–.101; *P=*.02). Additionally, the frequency of doctor visits had a positive effect on the intention to use online appointment systems and age had a negative effect on digital anamnesis tools. Compared with respondents who had not visited a doctor in the last 12 months, all frequency groups showed a positive effect (1 visit in the last 12 months: B=0.783, SE 0.285; β=0.213; *P=*.006; 2-3 visits: B=0.698, SE 0.267; β=.233; *P=*.009; >3 visits: B=0.690, SE 0.270; β=.224; *P=*.01). Compared with the age group of 18-30 years, there were negative effects for the age groups of 51-65 years (B=–0.476, SE 0.235; β=–.125; *P=*.04) and >65 years (B=–0.614, SE 0.239; β=–.158; *P=*.01).

The size of the residence city, region, and type of insurance did not affect the intention to use any digital use cases. Remarkably, Welch ANOVA found that digital literacy was dependent on age (Welch *F*_3,968.29_=53.441; *P<*.001).

## Discussion

### Principal Findings

This research paper aimed to compare the different technology acceptance factors of patients for key digital use cases in family medicine and to support family doctors in selecting and implementing digital use cases. Our results indicated that the degree of digitalization of family practice is not critically important for patients. Additionally, our results fully supported hypothesis A, highlighting that different use cases have differences in awareness, availability, and actual usage among patients. Further, we validated hypothesis B, showing that different use cases are affected by different technology acceptance factors. Nevertheless, our results only partially supported hypothesis C as digital literacy had a significant effect on the intention to use for all use cases.

Thus, the varying relative importance of technology acceptance factors for each digital use case emphasizes the need for a nuanced selection and implementation approach of digital use cases for family practice. Simultaneously, the partial rejection of different effects of sociodemographic variables could imply that our recommendations can be applied in different sociodemographic contexts.

### Comparison With Prior Work

We introduced a systematic assessment of technology acceptance for online booking platforms and digital anamnesis tools as existing studies concentrated only on video consultation platforms and electronic health records. Hence, we could only compare our results to prior work on these 2 use cases.

#### Receiving Medical Consultations via Video

The strongest effect of *performance expectancy* has been validated in various studies [18,49]. However, the literature indicates mixed results for the effect of *effort expectancy* and *social influence* on the intention to use video consultation platforms. For example, Schmitz et al [18] rejected the effect of *social influence*, claiming that medical appointments are highly personal, and thus, the fact of undergoing a medical treatment is not subjective to the opinions of others, regardless of a virtual or physical setting. However, our findings showed that the mode of the consultation might be affected by other opinions. This is supported by Esber et al [17], who confirmed the significance of *social influence* on the acceptance of video consultations, and Mueller et al [4], who stressed the importance of social cues to drive the technology acceptance of preusers.

Additionally, we found support for our rejection of the effect of *facilitating condition* on the intention to use video consultations in previous work [18,39,49,50]. Schmitz et al [18] and Brooks [51] mention that video consultation technologies are comparable to already widespread technologies, such as FaceTime, and thus, people are already well equipped for their use in a medical setting.

Surprisingly, we rejected the effect of *perceived privacy and security* on the intention to use video consultations but accepted this effect for other use cases. This contrasts with the findings of Zobair et al [52] and Schmitz et al [18] who highlighted the effect of perceived security on the intention to use telemedicine or telehealth in Bangladesh and Germany. Viana Pereira et al [39] postulated that doctors could also provide advice on general health topics via video and thus would not cause confidentiality concerns among patients. Nevertheless, we argued that the neglect of the importance of *perceived privacy and security* for patients requires further validation.

#### Storing Personal Medical Information via Electronic Health Records

*Performance expectancy* was constantly confirmed as a significant predictor of intention to use [16,20,40,53-63]. Additionally, we found evidence for positive effects of *effort expectancy* [16,20,53,55-57,62] and *facilitating condition* [20,60] on intention to use. Nevertheless, our findings contradict those of Tavares and Olivera [59] who suggested that early adopters do not perceive obstacles in using electronic health records due to their higher cognitive ability and experience with both technological complexities and IT infrastructure. This is further supported by Aydin and Kumru [40] who rejected *facilitating condition* as a predictor of the intention to use electronic medical records for Gen-Z university students. We explain this discrepancy with our more diverse sample, which we believe is less biased toward high cognitive ability and technological experience.

Our findings of a positive effect of *social influence* confirm the findings of Tavares et al [16] who illustrated inadequate promotion activities as a possible reason for the failed implementation of a comparable health record technology [64]. Nevertheless, Abd-Alrazaq et al [20] identified *social influence* as a nonsignificant predictor for behavioral intention, explaining that usage is voluntary and thus less objective to *social influence*. We hypothesized that the ongoing discussion on the transition from an opt-in to an opt-out model in Germany (default mandatory use of electronic health records) could partially explain the significance of *social influence* in our research [10].

Further studies that assessed comparable constructs of *perceived security and privacy* confirmed the positive effect on the intention to use electronic health records [20,40,53,55,56].

### Effect of Sociodemographic Variables on the Intention to Use Digital Use Cases

Except for digital literacy, we found no clear pattern of the effect of sociodemographic variables on the intention to use a specific digital use case. The vital role of digital literacy in technology acceptance is in line with research that postulates a digital divide of patient groups in engaging in digital use cases [65]. However, this role of digital literacy necessitates further differentiation. First, the participants in our study might be rather digitally savvy, and thus, this effect might be overgeneralized. Second, we acknowledge different magnitudes of this effect across use cases, suggesting that inherent characteristics of use cases (eg, technical complexity) might impact the role of digital literacy in technology acceptance. In identifying digitally literate patients, our findings showed a negative effect of age on digital literacy, with further evidence in other studies [66].

Our study found no effect of the residence city size on patients’ intention to use video consultations. This is particularly interesting as, for example, receiving video consultations is seen as a means to ensure adequate treatment opportunities in rural areas, with Kane and Gillis [67] highlighting that the use of telemedicine by physicians is higher in nonmetropolitan areas than in metropolitan areas in the United States. On the other hand, McGrail et al [68] noted no difference in the offering of telemedicine between rural and urban settings in Canada. This contradiction indicates that the effect of the residence city size on intention to use or actual usage depends on further factors (eg, the potential difference in the digital literacy of people living in rural vs metropolitan areas or the lack of availability of video consultation offerings in some regions).

### Measures for Family Doctors to Increase Patient Usage of Digital Use Cases

#### Importance of the Digital Maturity of Family Practices for Patients

Family doctors should carefully assess whether investing in digital use cases is adequate to attract and sustain patients. Our results showed that the level of digitalization of family practices was not a vital criterion for patients when choosing a doctor. This confirms the findings of Kuruoglu et al [69] and Khatami et al [70] who did not mention the degree of digitalization as a selection criterion for patients.

Nevertheless, our results showed that patients with higher digital literacy had greater importance for the degree of digitalization when selecting a family practice. Thus, digital investment may be more vital for family doctors treating more digitally literate patients. Hence, family doctors who refuse to invest in digital technology could lose these patient groups to family doctors who invest in digital technology.

#### Selection of Digital Use Cases

Family doctors should consider patient demand in the selection of digital use cases. Our study highlights the attractiveness of use cases for patients by comparing offering and actual usage rates. According to this approach, scheduling doctor appointments online appeared to be the most attractive digital use case in Germany in 2023, followed by providing personal information before consultation digitally (digital anamnesis). Video consultation was the least favorable use case for patients. Interestingly, a 2023 study on the digital offerings of family doctors in Germany indicated that video consultations were nearly 4 times more often offered than digital anamnesis tools [71]. Nevertheless, we are aware that patient demand is only 1 factor for offering use cases [13], and further patient-specific and use case–specific barriers might bias this attractivity assessment. We excluded electronic health records as family doctors in Germany must document patient treatment within the respective electronic health record of the patient (“Elektronische Patientenakte” [10]). Hence, family doctors do not select this digital use case voluntarily.

#### Implementation of Digital Use Cases

To incentivize usage among patients, family doctors should adopt an implementation approach for each use case, given that we showed the varying importance of technology acceptance factors in each use case. Nevertheless, we identified 3 factors that affected the intention to use for all use cases, and these should always be considered by family doctors.

First, doctors should prioritize the value addition of the digital use case to the patient. This is especially important for video consultations. The focus on value addition to drive adoption aligns with other academic papers [18,49] that stress the ability of doctors to communicate the added value to the patient. Hence, family doctors should receive adequate training or communication material from providers to explain the added value to patients effectively.

Second, family doctors should assess the importance of an intuitive interface for solutions as *effort expectancy* had a significant effect across all use cases. Nevertheless, the relative importance of this factor varied by use case. Since we believe that *effort expectancy* is dependent on the design and user experience of solutions and family doctors have limited control over these, the correct initial selection of a solution provider is vital.

Third, family practices should consider the digital literacy of their patients as it is a predictor of the intention to use across all use cases. We acknowledge that the digital literacy of family doctors affects their own technology adoption [72]. Hence, we believe that not only family doctors themselves should ensure that patients with low digital literacy are not excluded from using digital use cases.

Further considerations for family doctors depend on the respective digital use case. To increase the use of video consultations, family doctors should additionally consider endorsements from testimonials or other influencing bodies. Surprisingly, we showed no effect of *perceived privacy and security* on the intention to use video consultations. This could indicate that this factor might be neglectable for family doctors; however, further validation is needed. On the contrary, *perceived privacy and security* is important for online booking platforms. Therefore, family doctors should articulate how online booking platforms ensure the privacy of end users and how platforms communicate about this. Additionally, *perceived privacy and security* is the second most important factor to drive the use of electronic health records. Therefore, family doctors should focus on whether patients feel their data are protected. While this factor is also to be considered for digital anamnesis tools, having an easy-to-use solution here is more important than for electronic health records.

### Limitations and Further Research

To the best of our knowledge, our web-based survey with 1880 included participants is one of the largest recent studies assessing technology acceptance in a health care setting. By comparing 4 distinct use cases, we provided a new nuanced assessment and avoided attitude generalization. Contrary to studies that only included users or nonusers of a digital use case [20,54], our research included both users and nonusers in a family medicine context.

Nevertheless, we acknowledge that our study had some limitations. First, due to our voluntary and web-based survey approach, our sample might be biased toward participants with higher digital literacy and higher interest in digital health topics. This excluded patients with no access to the internet. However, we believe our sample is appropriate as digital use cases require internet access and our sample includes these potential users. Additionally, we tried to minimize bias by distributing physical questionnaires in family practices; however, responses to distributed flyers were limited. Second, replicating the same construct with the exact wordings for each use case potentially did not address use case specifics and thus increased the risk of ambiguity of items. For example, an electronic health record is not offered by the family doctor but rather by the insurance provider. In addition, the questionnaire was translated into German for the first time, which might explain the presence of a few low Cronbach α values. Dropping items would have significantly increased the Cronbach α values; however, to ensure comparability with the original questionnaire, we refrained from doing so. For example, for the worst Cronbach α (scheduling doctor appointments online: *facilitating condition*), only 1 item showed a low correlation with the other items. Furthermore, we did not have access to data validating the offer rates and usage rates of digital use cases by the participants but rather relied on the subjective inputs of the respondents. Nevertheless, as we offered the response options “I don’t know” and “Not specified,” we hypothesized high validity in the remaining responses with “Yes” or “No” answer entries. In addition, we did not account for prior experience, potentially not differentiating the distinct effects of expectations or past use on technology acceptance. Finally, we only looked at behavioral intention to use; however, various studies have highlighted a gap between intention and usage behavior, which is referred to as the intention-behavior gap [73]. Thus, our research did not predict the actual usage of digital use cases.

There are 3 areas for future research. First, it is vital to understand the technology acceptance of people with limited access to the internet (eg, people living in elderly homes) or low digital literacy and discuss measures on how to minimize the digital divide. Second, the effects of the “Digital Act (Digital-Gesetz)” of Germany [10] on usage by patients should be examined, particularly the mandatory implementation of electronic health records. Third, our research focused on Germany, and thus, the applicability of our findings to other countries could be discussed.

### Conclusion

Patients assign lower importance to the degree of digitalization of their family practices. Additionally, the usage of digital use cases remains low. Therefore, family doctors should carefully assess whether investments in digital use cases are adequate. Nevertheless, as the benefits of these solutions are established in the literature, we see future potential for higher adoption. For capturing this adoption potential, we believe in the importance of the role of family doctors owing to the established and trusted patient-doctor relationship. To leverage this role and increase patient usage, family doctors should align their selection and implementation criteria with our findings of the varying technology acceptance factors. We showed that *performance expectancy*, *effort expectancy*, and *digital literacy* have positive effects on the intention to use across all use cases, but further acceptance factors depend on the use case. To illustrate, family doctors should always emphasize the perceived added value for patients, whereas the neglect of privacy and security aspects for video consultation platforms requires further validation. Family doctors should always prioritize *performance expectancy*, while guides for the selection and implementation of digital tools might emerge for each digital use case.

## Appendix

Multimedia Appendix 1 Literature review (including the PRISMA-ScR [Preferred Reporting Items for Systematic Reviews and Meta-Analyses extension for Scoping Reviews] checklist).

Multimedia Appendix 2 Translated survey questionnaire.

Multimedia Appendix 3 CHERRIES (Checklist for Reporting Results of Internet E-Surveys).

Multimedia Appendix 4 Overview of regression coefficients for the regression models predicting the intention to use digital use cases.

## Abbreviations

CHERRIES: Checklist for Reporting Results of Internet E-Surveys
PRISMA-RR: Preferred Reporting Items for Systematic Reviews and Meta-Analyses extension for Rapid Reviews
PRISMA-ScR: Preferred Reporting Items for Systematic Reviews and Meta-Analyses extension for Scoping Reviews
UTAUT: Unified Theory of Acceptance and Use of Technology

## Data Availability

The datasets incorporated and examined during this research project are available from the corresponding author upon reasonable request and on consideration of restrictions associated with the ethics approval.
