# Supplementary material for: Patient Adoption of Digital Use Cases in Family Medicine and a Nuanced Implementation Approach for Family Doctors: Quantitative Web-Based Survey Study
Source: JMIR Form Res. 2025 Mar 5;9:e58867. doi: 10.2196/58867 (PMC11923474; doi:10.2196/58867)
Supplement: Multimedia Appendix 1 [file formative_v9i1e58867_app1.docx]

**Multimedia Appendix 1.** Literature review (including the PRISMA-ScR [Preferred Reporting Items for Systematic Reviews and Meta-Analyses extension for Scoping Reviews] checklist).

1. **Literature Review on Technology Acceptance of selected Digital Use Cases in Family Medicine**

### **Methods**

With respect to our research focus, we decided to conduct a rapid literature review, aligned to PRISMA-RR as well as recommendations given by Tricco et al. [1] and King et al. [2]. As PRISMA-RR is still under development, we adapted PRISMA guidelines (especially PRIMSA-ScR) to the constraints of our project. In our literature research, we screened three different databases (PubMed, Psychinfo, and Scopus) with a search strategy based on the connection of three building blocks via the Boolean Operator “AND”: 1) technology acceptance models, 2) patient views, and 3) the respective digital use cases. We limited our search results to being published in German or English from 2018 to 2023. Additionally, further relevant papers were included via backward citation-searching. In our abstract and full-time screening, we excluded studies that (1) were not peer-reviewed and with no access to the full text, (2) did not assess one of our four digital use cases in scope, (3) did not use a systematic technology acceptance model, (4) did not retrieve quantitative results or (5) did not focus on the perspective of the patients. The detailed criteria, including the search string, can be found in the checklist in Multimedia Appendix 1: PRISMA-ScR Checklist.

The research team agreed to not limit studies to a regional focus to highlight potential contextual differences in the technology acceptance criteria of patients. Nevertheless, as we focus in our paper on the family medicine context, we followed the definition of the World Organization of Family Doctors (WONCA) that the terms general practice and family medicine are identical and, among others, characterized as “normally the point of first medical contact within the health care system, providing open and unlimited access to its users, dealing with all health problems regardless of the age, sex, or any other characteristic of the person concerned” [3]. For consistency*,* we refer to the term “family doctors" in this paper. The results of the included studies were synthesized by assessing the effect of the variables used in our research model on intention to use/attitude towards the digital use case/ attitude to use.

### **Results**

The search strategy on patient technology acceptance of the four digital use cases resulted in 5,494 citations, of which 1,795 studies were narrowed down for abstract screening. After title and abstract screening regarding relevance and meeting pre-defined inclusion criteria, 139 records were sought for retrieval. As 25 articles did not allow for access to full texts, 114 articles were assessed for eligibility. Additionally, citation searching resulted in 13 results of which 11 reports could be assessed for eligibility. To conclude, using the identification of studies via databases as well as other methods, 29 studies were included in our literature review of our study. An overview of the screening process can be found in Figure 3.

*Figure 1. Flowchart for the literature review following PRISMA guidelines. Records removed for other reasons refer to removal of citations due to publication date and language exclusion criteria.*


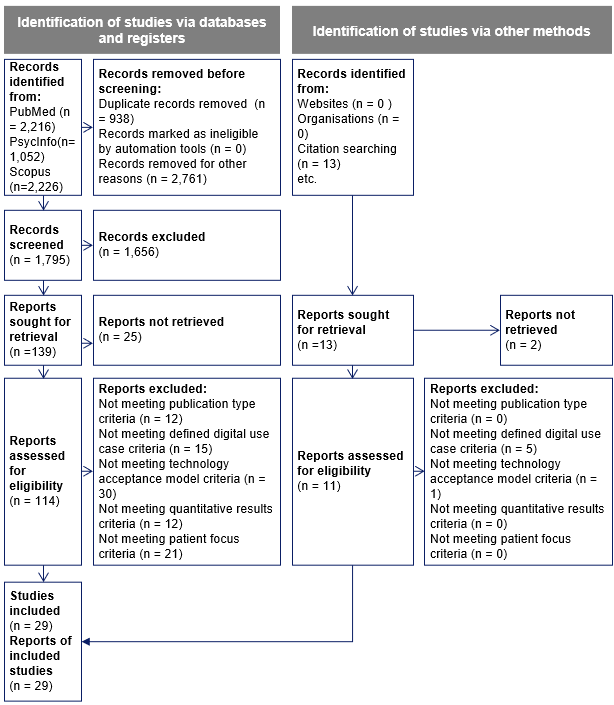


Our literature review yielded four insights. To the best of our knowledge, there are no studies assessing online-booking platforms and digital anamnesis tools with any technology acceptance model. 14 records (14/29, 48%) describe technology acceptance of electronic health records [4–17] and 15 (15/29, 52%) do so for video consultation platforms [18–32]. Second, only 3 studies (3/29, 10%) refer to Germany [10,20,27]. Third, our literature review showed mixed results regarding the effect of the described factors on technology acceptance (approximated by behavioral intention to use, actual usage, or attitude towards the digital use case). All but one of the included studies accepted the hypothesis that Performance Expectancy affects technology acceptance (28/29, 97%) [4–24,26–32], while no clear consensus can be found for the effect on intention to use for Effort Expectancy (accepted 15/28, 54%) [6–8,11,14,15,17–19,22,25,27–30], Social Influence (accepted 5/14, 36%) [8,15–17,27], and Facilitating Condition (accepted 3/12, 25%) [4,6,21]. Further, we found various evidence for an effect of Perceived Privacy and Security (accepted 7/8, 88% approximated by comparable variables in other studies) [6,11,14,16,20,29,30] which further strengthens our decision to include the Perceived Privacy and Security construct in our research model. Lastly, we did not find any paper comparing the technology acceptance of multiple digital health care use cases in one single paper.

### **Conclusion**

Based on our previous literature review, we add to the academic discourse with our findings for three reasons. First, most of the studies are not exclusive to Germany. Second, the heterogeneity of significant factors in determining technology acceptance in our literature review further strengthens our research gap that technology acceptance is different for specific use cases, however to the best of our knowledge, no paper has yet compared the technology acceptance of patients for several technologies in one paper. Lastly, we introduce a systematic technology acceptance assessment for online-booking platforms and digital anamnesis tools as we only found single-use case assessments for video consultation platforms and electronic health records.

1. **Completed PRISMA-ScR (Preferred Reporting Items for Systematic Reviews and Meta-Analyses extension for Scoping Reviews) checklist.**

| SECTION | ITEM | PRISMA-ScR CHECKLIST ITEM | REPORTED IN SECTION |
| --- | --- | --- | --- |
| TITLE | | | |
| Title | 1 | Identify the report as a scoping review. | n/a |
| ABSTRACT | | | |
| Structured summary | 2 | Provide a structured summary that includes (as applicable): background, objectives, eligibility criteria, sources of evidence, charting methods, results, and conclusions that relate to the review questions and objectives. | **Background:** Several studies in health care have examined patients’ overall attitude toward digitalization as well as for specific use cases. However, these studies fail to provide a comparison of patient acceptance criteria between inherent different digital use cases in family medicine.  **Objective:** To address this research gap, this paper aims to assist family doctors in selecting digital use cases by comparing their underlying patient adoption factors and in driving usage of these use cases by elaborating a differentiated implementation approach.  **Sources of Evidence:** In our literature research, we screened three different databases (PubMed, Psychinfo, and Scopus) with a search strategy based on the connection of three building blocks via the Boolean Operator “AND”: 1) technology acceptance models, 2) patient views, and 3) the respective digital use cases. The search was initially conducted in March 2023 and updated in September 2023. We limited our search results to being published in German or English from 2018 to 2023. Duplicates were manually removed. Additionally, further relevant papers were included via citation-searching.  **Results:** The search strategy resulted in 5.494 citations**.** Including five added papers via citation searching, eventually 29 studies were incorporated for analysis. To the best of our knowledge, there are no studies assessing online-booking platforms and digital anamnesis tools with any technology acceptance model. 14 records (14/29, 48%) describe technology acceptance of electronic health records and 15 do so for video consultation platforms. Second, only 3 studies (3/29, 10%) refer to patients in Germany. Third, our literature review showed mixed results regarding the effect of the factors of our research model on technology acceptance. Lastly, we did not find any paper comparing the technology acceptance of multiple digital health care use cases in one single paper.  **Conclusion**: The heterogeneity of significant factors in determining technology acceptance in our literature review further strengthens our research gap that technology acceptance is different for specific use cases. |
| INTRODUCTION | | | |
| Rationale | 3 | Describe the rationale for the review in the context of what is already known. Explain why the review questions/objectives lend themselves to a scoping review approach. | Overall adoption rates of digital health care use cases by both patients and family doctors are still at low levels. In assessing technology acceptance, we premise that differentiation and comparison between use cases might offer fruitful insights, as we hypothesize that there are inherent structural differences between digital use cases (e.g., degree of voluntariness of use for patients or degree of dealing with sensitive medical information). In doing so, we add to the academic discourse since there are several studies in health care examining patients’ overall digital attitude towards digitalization as well as for specific use cases however, these studies fail to provide a nuanced assessment, and comparison of patient acceptance criteria between different digital use cases in family medicine. This differentiation is particularly vital as potentially diverse patient technology acceptance criteria per use case might emerge, and therefore, a differentiated approach to drive adoption might be needed. |
| Objectives | 4 | Provide an explicit statement of the questions and objectives being addressed with reference to their key elements (e.g., population or participants, concepts, and context) or other relevant key elements used to conceptualize the review questions and/or objectives. | This paper aims to support family doctors in (i) selecting digital use cases by showcasing the relative importance of the digital use case for patients and their underlying adoption factors, as well as in (ii) driving usage of the digital use case by highlighting the need for a differentiated implementation approach based on different patient acceptance criteria per digital use case. In doing so, we focus on patients because if end-users do not intend to use the solution, family doctors are less likely to implement these patient-facing use cases |
| METHODS | | | |
| Protocol and registration | 5 | Indicate whether a review protocol exists; state if and where it can be accessed (e.g., a Web address); and if available, provide registration information, including the registration number. | We did not compile a review protocol as our rapid literature review was only part of a study design consisting of multiple methodical approaches. |
| Eligibility criteria | 6 | Specify characteristics of the sources of evidence used as eligibility criteria (e.g., years considered, language, and publication status), and provide a rationale. | We limited our search results to being published in German or English from 2018 to 2023 to incorporate the latest academic insights regarding technology acceptance in a health care setting. Additionally, further relevant papers were included via citation-searching to not miss any further important academic findings. |
| Information sources | 7 | Describe all information sources in the search (e.g., databases with dates of coverage and contact with authors to identify additional sources), as well as the date the most recent search was executed. | In our literature research, we screened three different databases (PubMed, Psychinfo, and Scopus) with a search strategy based on the connection of three building blocks via the Boolean Operator “AND”: 1) technology acceptance models, 2) patient views, and 3) the respective digital use cases. The initial search was conducted in March 2023 and updated in September 2023. Identification via other methods happened on a case-by-case basis in September and October 2023. |
| Search | 8 | Present the full electronic search strategy for at least 1 database, including any limits used, such that it could be repeated. | In our literature research, we screened three different databases (PubMed, Psychinfo, and Scopus) with a search strategy based on the connection of three building blocks via the Boolean Operator “AND”: 1) technology acceptance models, 2) patient views, and 3) the respective digital use cases. All three databases were searched with the same search strategy based on title- and abstract search. In the following, the search string for PubMed is illustrated:  ((( ("Technology Acceptance Model"[Title/Abstract]) OR ("TAM"[Title/Abstract]) OR ("UTAUT"[Title/Abstract]) OR ("UTAUT*"[Title/Abstract]) OR ("Unified Theory of Acceptance and Use of Technology"[Title/Abstract]) OR ("Acceptance"[Title/Abstract]) OR ("Technology Acceptance Prediction"[Title/Abstract]) OR ("Technology Acceptance"[Title/Abstract]) OR ("Intention to use"[Title/Abstract]) OR ("Perceived usefulness"[Title/Abstract]) OR ("Perceived ease of use"[Title/Abstract]) OR ("Technology Adoption"[Title/Abstract]) OR ("Performance Expectancy"[Title/Abstract]) OR ("Effort Expectancy"[Title/Abstract]) OR ("Behavioural Intention" Acceptance[Title/Abstract]) OR ("Behavioral Intention" Acceptance[Title/Abstract]) OR ("Technology Acceptance Models"[Title/Abstract]) OR ("Behavior Intention" Acceptance[Title/Abstract]) )) **AND** (( ("Patient*"[Title/Abstract]) OR ("User*"[Title/Abstract]) OR ("Patient view"[Title/Abstract]) OR ("User view"[Title/Abstract]) OR ("Enduser"[Title/Abstract]) OR ("End-user"[Title/Abstract]) OR ("patient-perspective"[Title/Abstract]) OR ("patient-perceived"[Title/Abstract]) OR ("End User"[Title/Abstract]) OR ("patient-view"[Title/Abstract]) OR ("user-view"[Title/Abstract]) )) **AND** (( ("anamnesis"[Title/Abstract]) OR ("appointment scheduling"[Title/Abstract]) OR ("digital anamnesis"[Title/Abstract]) OR ("digital medical interview assistant"[Title/Abstract]) OR ("digital medical interview assistants"[Title/Abstract]) OR ("digital patient record"[Title/Abstract]) OR ("digital patient records"[Title/Abstract]) OR ("direct scheduling of patient appointments"[Title/Abstract]) OR ("e-appointment scheduling"[Title/Abstract]) OR ("electronic health care record"[Title/Abstract]) OR ("electronic health care records"[Title/Abstract]) OR ("Electronic Health Record"[Title/Abstract]) OR ("Electronic Health Records"[Title/Abstract]) OR ("electronic healthcare record"[Title/Abstract]) OR ("electronic healthcare records"[Title/Abstract]) OR ("Electronic medical record"[Title/Abstract]) OR ("Electronic medical records"[Title/Abstract]) OR ("electronic patient record"[Title/Abstract]) OR ("electronic patient records"[Title/Abstract]) OR ("Health Data Collection"[Title/Abstract]) OR ("Health History Tool"[Title/Abstract]) OR ("Health History-Taking"[Title/Abstract]) OR ("health registration system"[Title/Abstract]) OR ("Medical History-Taking"[Title/Abstract]) OR ("medical record"[Title/Abstract]) OR ("medical records"[Title/Abstract]) OR ("online appointment scheduling"[Title/Abstract]) OR ("online scheduling"[Title/Abstract]) OR ("patient anamnesis"[Title/Abstract]) OR ("personal digital assistant"[Title/Abstract]) OR ("personal digital assistants"[Title/Abstract]) OR ("personal health record*"[Title/Abstract]) OR ("remote consultation"[Title/Abstract]) OR ("remote consultations"[Title/Abstract]) OR ("self-anamnesis"[Title/Abstract]) OR ("self-scheduling"[Title/Abstract]) OR ("teleconsultations"[Title/Abstract]) OR ("tele-consultations"[Title/Abstract]) OR ("Telehealth"[Title/Abstract]) OR ("tele-health"[Title/Abstract]) OR ("Telemedicine"[Title/Abstract]) OR ("video consultation"[Title/Abstract]) OR ("video consultations"[Title/Abstract]) OR ("videoconsultation"[Title/Abstract]) OR ("video-consultation"[Title/Abstract]) OR ("video-consultations"[Title/Abstract]) OR ("Virtual doctor appointment*"[Title/Abstract]) OR ("Web-based appointment scheduling"[Title/Abstract]) OR ("Web-based scheduling"[Title/Abstract]) ))) |
| Selection of sources of evidence | 9 | State the process for selecting sources of evidence (i.e., screening and eligibility) included in the scoping review. | In our abstract and full-time screening, we excluded studies that (1) were not peer-reviewed and with no access to the full text to ensure the high quality of our research. Further, to ensure comparability to our research we excluded reports that (2) did not assess one of our four digital use cases in scope (3) did not use a systematic technology acceptance (4) did not retrieve quantitative results (5) and did not focus on the perspective of the patients. |
| Data charting process | 10 | Describe the methods of charting data from the included sources of evidence (e.g., calibrated forms or forms that have been tested by the team before their use, and whether data charting was done independently or in duplicate) and any processes for obtaining and confirming data from investigators. | JB independently collected characteristics and findings of studies relevant to our research object in a single sheet. To ensure comparability among different wordings of different research papers, JB manually harmonized inputs of included studies. |
| Data items | 11 | List and define all variables for which data were sought and any assumptions and simplifications made. | JB sought data for the following variables of our research model, Performance Expectancy, Effort Expectancy, Social Influence, Facilitating Condition and Perceived Privacy and Security. In particular, we searched for the effect of these variables on the dependent variable intention to use a particular use case. We simplified the dependent variable to also include comparable constructs (e.g., attitude towards the digital use case or attitude to use) as well as for the added UTAUT construct of Perceived Privacy and Security, being aware of potential limitations of this approach. To ensure comparability of digital use cases researched in other papers to the digital use cases in our scope, we followed the use case definitions illustrated in the chapter “Literature Review”. These definitions guided our decision-making to ensure the comparability of our research to prior academic work. Additionally, the following additional information was retrieved per included study: Author, title, journal, publication year, identifier Use case, location, sample group, sample size, research design, technology acceptance model used, |
| Critical appraisal of individual sources of evidence | 12 | If done, provide a rationale for conducting a critical appraisal of included sources of evidence; describe the methods used and how this information was used in any data synthesis (if appropriate). | As the rapid literature review was part of a multifaced study design, we did not conduct a dedicated critical appraisal due to resource constraints. Further, our primary goal was to highlight the effects of the described variables on the intention to use a particular use case and not to assess the quality of previous research undertaken. |
| Synthesis of results | 13 | Describe the methods of handling and summarizing the data that were charted. | The results of the included studies were synthesized by assessing the effect of the variables used in our research model (Performance Expectancy, Effort Expectancy, Social Influence, Facilitating Condition and Perceived Privacy and Security) on intention to use/attitude towards the digital use case/ attitude to use. |
| RESULTS | | | |
| Selection of sources of evidence | 14 | Give numbers of sources of evidence screened, assessed for eligibility, and included in the review, with reasons for exclusions at each stage, ideally using a flow diagram. | The search strategy resulted in 5.494 citations, of which 1.795 studies were narrowed down for abstract screening. After title and abstract screening regarding relevance and meeting pre-defined inclusion criteria, 139 records were sought for retrieval. As 25 articles did not allow for access to full texts, 114 articles were assessed for eligibility. Additionally, citation searching resulted in 13 results of which 11 reports could be assessed for eligibility. To conclude, using the identification of studies via databases as well as other methods, 29 studies were included in our literature review of our study. An overview of the screening process can be found in Figure 3. |
| Characteristics of sources of evidence | 15 | For each source of evidence, present characteristics for which data were charted and provide the citations. | Please refer to chapter 1.1.  The sheet incorporating the relevant findings and characteristics per source of evidence can be retrieved from JB upon request. |
| Critical appraisal within sources of evidence | 16 | If done, present data on critical appraisal of included sources of evidence (see item 12). | n/a |
| Results of individual sources of evidence | 17 | For each included source of evidence, present the relevant data that were charted that relate to the review questions and objectives. | Please refer to chapter 1.1.  The sheet incorporating the relevant findings and characteristics per source of evidence can be retrieved from JB upon request. |
| Synthesis of results | 18 | Summarize and/or present the charting results as they relate to the review questions and objectives. | Our literature review yielded four insights. To the best of our knowledge, there are no studies assessing online-booking platforms and digital anamnesis tools with any technology acceptance model. 14 records (14/29, 48%) describe technology acceptance of electronic health records and 15 (15/29, 52%) do so for video consultation platforms. Second, only 3 studies (3/29, 10%) refer to patients in Germany. Third, our literature review showed mixed results regarding the effect of the described factors on technology acceptance (approximated by behavioral intention to use, actual usage, or attitude towards the digital use case). All but one of the included studies accepted the hypothesis that Performance Expectancy affects technology acceptance (28/29, 97%) while no clear consensus can be found for the effect on intention to use for Effort Expectancy (accepted 15/28, 54%), Social Influence (accepted 5/14, 36%), and Facilitating Condition (accepted 3/12, 25%). Further, we found various evidence for an effect of Perceived Privacy and Security (accepted 7/8, 88% approximated by comparable variables in other studies) –which further strengthens our decision to include the Perceived Privacy and Security construct in our research model. Lastly, we did not find any paper comparing the technology acceptance of multiple digital health care use cases in one single paper. |
| DISCUSSION | | | |
| Summary of evidence | 19 | Summarize the main results (including an overview of concepts, themes, and types of evidence available), link to the review questions and objectives, and consider the relevance to key groups. | To the best of our knowledge, there are no studies assessing online-booking platforms and digital anamnesis tools with any technology acceptance model and overall, only a few studies refer to the German context. Further, our literature review strengthens our research gap that technology acceptance factors are different for specific use cases except for Performance Expectancy) as we identified heterogeneity of significant factors in determining technology acceptance for different use cases. |
| Limitations | 20 | Discuss the limitations of the scoping review process. | Different wordings for potentially same use cases or variables (e.g., Perceived Privacy and Security) could potentially have biased the selection of included reports. JB tried to mitigate this risk as established definitions of the digital use cases have been followed and studies have been screened in light of these definitions. Furthermore, the applied inclusion and exclusion criteria might limit the number of studies screened and thus we might have not included all of the relevant papers, nevertheless, we tried to mitigate this risk by applying citation searching. Additionally, no second researcher validated our screening processes as the rapid literature review conducted was only part of a multi-faced study design. |
| Conclusions | 21 | Provide a general interpretation of the results with respect to the review questions and objectives, as well as potential implications and/or next steps. | Based on our previous literature review, we identified three research gaps that we aimed to address with our paper. First, most of the studies are not exclusive to Germany thus further research is needed to consider the specific German context. Second, the heterogeneity of significant factors in determining technology acceptance in our literature review further strengthens our research gap that technology acceptance is different for specific use cases, however to the best of our knowledge, no paper has yet compared the technology acceptance of patients for several technologies in one paper. Lastly, we introduce a systematic technology assessment for online-booking platforms and digital anamnesis tools as we only found single-use case assessments for video consultation platforms and electronic health records. |
| FUNDING | | | |
| Funding | 22 | Describe sources of funding for the included sources of evidence, as well as sources of funding for the scoping review. Describe the role of the funders of the scoping review. | JB did not get any funding for this research project as this is part of a doctoral thesis at Witten/Herdecke University. |

References

1. Tricco AC, Zarin W, Antony J, Hutton B, Moher D, Sherifali D, Straus SE. An international survey and modified Delphi approach revealed numerous rapid review methods. J Clin Epidemiol 2016;70:61-67. doi:10.1016/j.jclinepi.2015.08.012

2. King VJ, Stevens A, Nussbaumer-Streit B, Kamel C, Garritty C. Paper 2: Performing rapid reviews. Systematic Reviews 2022;11(1):151. doi:10.1186/s13643-022-02011-5

3. WONCA. The European Definition of General Practice / Family Medicine 2023 URL: https://www.woncaeurope.org/file/41f61fb9-47d5-4721-884e-603f4afa6588/WONCA_European_Definitions_2_v7.pdf [accessed 2024-01-12].

4. Keung KL, Lee C, Ng KK, Leung S, Choy K. An Empirical Study on Patients' Acceptance and Resistance Towards Electronic Health Record Sharing System: A Case Study of Hong Kong. International Journal of Knowledge and Systems Science 2018;9:1-27. doi:10.4018/IJKSS.2018040101

5. Alharbey R. Integrative adoption model for personal health records: A structural equation modeling approach. International Journal of ADVANCED AND APPLIED SCIENCES 2022;9:117-127. doi:10.21833/ijaas.2022.01.014

6. Abd-Alrazaq A, Bewick BM, Farragher T, Gardner P. Factors Affecting Patients’ Use of Electronic Personal Health Records in England: Cross-Sectional Study. J Med Internet Res 2019;21(7):e12373. doi:10.2196/12373

7. Hoogenbosch B, Postma J, Man-van Ginkel JM de, Tiemessen N am, van Delden JJ, van Os-Medendorp H. Use and the Users of a Patient Portal: Cross-Sectional Study. J Med Internet Res 2018;20(9):e262. PMID:30224334

8. Tavares J, Goulão A, Oliveira T. Electronic Health Record Portals adoption: Empirical model based on UTAUT2. Inform Health Soc Care 2018;43(2):109-125. doi:10.1080/17538157.2017.1363759

9. Tavares J, Oliveira T. New Integrated Model Approach to Understand the Factors That Drive Electronic Health Record Portal Adoption: Cross-Sectional National Survey. J Med Internet Res 2018;20(11):e11032. PMID:30455169

10. Niemöller S, Hübner U, Egbert N, Babitsch B. How to Access Personal Health Records? Measuring the Intention to Use and the Perceived Usefulness of Two Different Technologies: A Randomised Controlled Study. Stud Health Technol Inform 2019;267:197-204. PMID:31483273

11. Abd-Alrazaq A, Alalwan AA, McMillan B, Bewick BM, Househ M, AL-Zyadat AT. Patients’ Adoption of Electronic Personal Health Records in England: Secondary Data Analysis. J Med Internet Res 2020;22(10):e17499. doi:10.2196/17499

12. Agrawal L, Ndabu T, Mulgund P, Sharman R. Factors Affecting the Extent of Patients' Electronic Medical Record Use: An Empirical Study Focusing on System and Patient Characteristics. J Med Internet Res 2021;23(10):e30637. PMID:34709181

13. Razmak J, Bélanger C. Using the technology acceptance model to predict patient attitude toward personal health records in regional communities. Information Technology & People 2018;31:0. doi:10.1108/ITP-07-2016-0160

14. Alsyouf A, Lutfi A, Alsubahi N, Alhazmi FN, Al-Mugheed K, Anshasi RJ, Alharbi NI, Albugami M. The Use of a Technology Acceptance Model (TAM) to Predict Patients' Usage of a Personal Health Record System: The Role of Security, Privacy, and Usability. International Journal of Environmental Research and Public Health 2023;20(2). PMID:36674105

15. Liu J, Gong X, Weal M, Dai W, Hou S, Ma J. Attitudes and associated factors of patients' adoption of patient accessible electronic health records in China - A mixed methods study. Digit Health 2023;9:20552076231174101. PMID:37188077

16. Aydin G, Kumru S. Paving the way for increased e-health record use: elaborating intentions of Gen-Z. Health Systems 2023;12(3):281-298. doi:10.1080/20476965.2022.2129471

17. Hassan I, Murad M, El-Shekeil I, Liu J. Extending the UTAUT2 Model with a Privacy Calculus Model to Enhance the Adoption of a Health Information Application in Malaysia. Informatics 2022;9:31. doi:10.3390/informatics9020031

18. Hsieh H-L, Lai J-M, Chuang B-K, Tsai C-H. Determinants of Telehealth Continuance Intention: A Multi-Perspective Framework. Healthcare (Basel) 2022;10(10). PMID:36292485

19. Alexandra S, Handayani PW, Azzahro F. Indonesian hospital telemedicine acceptance model: the influence of user behavior and technological dimensions. Heliyon 2021;7(12):e08599. doi:10.1016/j.heliyon.2021.e08599

20. Schmitz A, Díaz-Martín AM, Yagüe Guillén MJ. Modifying UTAUT2 for a cross-country comparison of telemedicine adoption. Comput Human Behav 2022;130:107183. PMID:35017788

21. Chan ZY, Lim CF, Leow JL, Chium FY, Lim SW, Tong CHM, Zhou JJX, Tsi MMY, Tan RYC, Chew LST. Using the technology acceptance model to examine acceptance of telemedicine by cancer patients in an ambulatory care setting. Proceedings of Singapore Healthcare 2022;31:20101058221104578. doi:10.1177/20101058221104578

22. Su Y-Y, Huang S-T, Wu Y-H, Chen C-M. Factors Affecting Patients' Acceptance of and Satisfaction with Cloud-Based Telehealth for Chronic Disease Management: A Case Study in the Workplace. Appl Clin Inform 2020;11(2):286-294. PMID:32294772

23. Baudier P, Kondrateva G, Ammi C, Chang V, Schiavone F. Patients' perceptions of teleconsultation during COVID-19: A cross-national study. Technol Forecast Soc Change 2021;163:120510. PMID:33318716

24. Amin R, Hossain MA, Uddin MM, Jony MTI, Kim M. Stimuli Influencing Engagement, Satisfaction, and Intention to Use Telemedicine Services: An Integrative Model. Healthcare (Basel) 2022;10(7). PMID:35885854

25. Holtz B, Mitchell K, Hirko K, Ford S. Using the Technology Acceptance Model to Characterize Barriers and Opportunities of Telemedicine in Rural Populations: Survey and Interview Study. JMIR Form Res 2022;6(4):e35130. PMID:35436207

26. Viana Pereira F, Tavares J, Oliveira T. Adoption of video consultations during the COVID-19 pandemic. Internet Interv 2023;31:100602. PMID:36694630

27. Esber A, Teufel M, Jahre L, Schmitten J in der, Skoda E-M, Bäuerle A. Predictors of patients' acceptance of video consultation in general practice during the coronavirus disease 2019 pandemic applying the unified theory of acceptance and use of technology model. Digit Health 2023;9:20552076221149317. PMID:36815005

28. Anil Kumar Vaidhyam S, Huang K-T. Social Determinants of Health and Patients' Technology Acceptance of Telehealth During the COVID-19 Pandemic: Pilot Survey. JMIR Hum Factors 2023;10:e47982. PMID:37934556

29. van Houwelingen CT, Ettema RG, Antonietti MG, Kort HS. Understanding Older People's Readiness for Receiving Telehealth: Mixed-Method Study. J Med Internet Res 2018;20(4):e123. PMID:29625950

30. An MH, You SC, Park RW, Lee S. Using an Extended Technology Acceptance Model to Understand the Factors Influencing Telehealth Utilization After Flattening the COVID-19 Curve in South Korea: Cross-sectional Survey Study. JMIR Med Inform 2021;9(1):e25435. PMID:33395397

31. Qi M, Cui J, Li X, Han Y. Perceived Factors Influencing the Public Intention to Use E-Consultation: Analysis of Web-Based Survey Data. J Med Internet Res 2021;23(1):e21834. PMID:33470934

32. Peixoto M, Ferreira J, Rodrigues de Oliveira L. Drivers for Teleconsultation Acceptance in Brazil: Patients’ Perspective during the COVID-19 Pandemic. Revista de Administração Contemporânea 2022;26. doi:10.1590/1982-7849rac2022210063.en
