# Supplementary material for: Patient Adoption of Digital Use Cases in Family Medicine and a Nuanced Implementation Approach for Family Doctors: Quantitative Web-Based Survey Study
Source: JMIR Form Res. 2025 Mar 5;9:e58867. doi: 10.2196/58867 (PMC11923474; doi:10.2196/58867)
Supplement: Multimedia Appendix 2 [file formative_v9i1e58867_app2.docx]

**Multimedia Appendix 2.** Translated survey questionnaire.

**Questionnaire: Translated into English Version**

*The German questionnaire used in this survey can be requested from the authors.*

| **Landing Page:**  Dear Sir or Madam  Thank you for your interest in this study.  As part of a scientific project at the Faculty of Health at University Witten/Herdecke, this survey examines patient **expectations and the use of four different digital applications in family practices**: **video consultations, online appointment booking, electronic health records and digital anamnesis**. We are particularly interested in your expectations and previous experiences with the above-mentioned applications. Additionally, we would also like to investigate the relationship to personal characteristics.  The aim of this research project is to analyze relevant factors concerning the acceptance of digital applications. These serve as a basis for describing different user groups and to derive specific recommendations for family doctors when implementing digital applications.  The survey is targeted at adult patients of family practices. We would therefore like to invite you to participate in a **10-to-15-minute**-long survey that asks about your personal experience with one of the above-mentioned digital applications in the family practice. To increase the value for academia, we ask you to **complete each question of the questionnaire**.  The ethics committee of the University Witten/Herdecke has positively evaluated this study under S-245/2022. The data collected is used for scientific research and cannot be traced back to you, so you remain anonymous. Information about your **rights as a participant and the data processing** of this survey can be found on the next page.  If you have any questions, please contact: julian.beerbaum@uni-wh.de  Thank you for your support. | | | | | | | | | | | | | | | | | | |
| --- | --- | --- | --- | --- | --- | --- | --- | --- | --- | --- | --- | --- | --- | --- | --- | --- | --- | --- |
| *Next page* | | | | | | | | | | | | | | | | | | |
| **Privacy policy and your rights as a participant**  **Checkbox**: I have been informed that the personal data of the study participants will also be processed as part of this study. I was informed for what purpose, to what extent, on what legal basis, how long this data will be stored and what rights the study participants have towards the responsible body with regard to their personal data. I have received a corresponding information letter (see privacy policy).  I am aware that my consent is voluntary and that I can revoke it at any time in writing or verbally without giving reasons and without any personal disadvantage. My data will then be completely deleted as long as it has not yet been anonymized. I will receive a message about this. | | | | | | | | | | | | | | | | | | |
| *Next page* | | | | | | | | | | | | | | | | | | |
| **Frequency of family doctor visits and legal age check**  To capture your experience with digital applications in the family practice, we would first like to know how often you have visited a family practice in the last year. For ethical reasons, we would also like to ensure that you have already reached the legal age when participating in the study.  How often have you visited your family practice in the last 12 months? *(Responded as a single-choice question)*   1. 0 2. 1 3. 2-3 4. >3   Did you reach legal age already? (Participants who answered “No” were not able to continue the survey; *Responded as a single-choice question*)   1. Yes 2. No | | | | | | | | | | | | | | | | | | |
| *Next page* | | | | | | | | | | | | | | | | | | |
| **Sociodemographic data and digital literacy**  In the following, we would like to collect information about your age, gender, place of residence and insurance status. In addition, we ask you to assess your own general affinity or competence in dealing with digital applications (e.g. in the use of online banking or online messenger services).  Which gender do you identify with? *(Responded as a single-choice question)*   1. Female 2. Male 3. Not specified 4. Divers, in fact: [Text field]   How old are you? *(Responded as a single-choice question)*   1. 18-30 years 2. 31-50 years 3. 51-65 years 4. Over 65 years 5. Not specified   In which federal state do you live? *(Responded as a single-choice question)*   1. Baden-Wuerttemberg 2. Bavaria 3. Berlin 4. Brandenburg 5. Bremen 6. Hamburg 7. Hessen 8. Mecklenburg-Vorpommern 9. Niedersachsen 10. North Rhine-Westphalia 11. Rheinland-Pfalz 12. Saarland 13. Saxony 14. Saxony-Anhalt 15. Schleswig-Holstein 16. Thuringia 17. Not specified   How old are you? *(Responded as a single-choice question)*   1. 18-30 years 2. 31-50 years 3. 51-65 years 4. Over 65 years 5. Not specified   What is the population size of your place of residence? *(Responded as a single-choice question)*   1. Less than 20,000 inhabitants 2. 20,001 to 200,000 inhabitants 3. More than 200,000 inhabitants 4. Not specified   Do you have private or statutory health insurance? *(Responded as a single-choice question)*   1. Private 2. Statutory 3. Not specified   How digitally savvy or competent do you generally consider yourself when using digital applications (e.g. in the use of online banking or online messenger services)? *(Responded as numeric input)*  Please rate your digital affinity with a number between 1-10 and enter it below using the following scale: 1="Not at all digital affine" - 10="Very digital affine")   1. I rate my digital affinity as: [Text field] | | | | | | | | | | | | | | | | | | |
| *Next page* | | | | | | | | | | | | | | | | | | |
| **Previous experience with digital applications in family practices**  In the following, we would like to examine your previous knowledge and experience with video consultations, online appointment booking, electronic health records and digital anamneses tools in a family practice. To do this, we ask you to first read the descriptions below carefully.  Video consultations  The video consultation includes online-supported patient care. Doctors can explain further treatment to their patients on screen, examine the healing process of a surgical wound or conduct a psychotherapeutic consultation. This means that patients do not have to come to the practice for every appointment. The doctor has to select a certified video service provider who will ensure that the video consultation runs smoothly and securely.  Online appointment booking  Online appointment booking is the process of booking and/or canceling appointments in family practices via the Internet. Online appointment booking enables the management of appointments at any time. This study refers to appointment management platforms that enable ad-hoc appointment confirmation.  Electronic health records  Health insurance companies provide their member with an electronic health record through an app free of charge. In the electronic health record, personal health information can be stored digitally in one place. This includes findings and diagnoses, but also treatment reports or information on recommended therapy. The electronic health record also offers digital versions of the vaccination record, maternity record, child examination booklet and dental bonus booklet. The user alone decides what information is included in the electronic health record and who is allowed to access it.  Digital anamnesis  Digital anamnesis refers to the approach of shifting part of the medical consultation to the preparation phase in order to make the consultation more efficient and goal-oriented. Software is used to collect the patient's medical history, complaints, concerns, worries and expectations, as well as administrative data. Formal processes, e.g., declarations of consent under the General Data Protection Regulation (GDPR), are also covered in this way. Patients work with the digital medical history system at home on their PC or smartphone or in the waiting room on a tablet.  Please indicate your agreement with the following statements for each digital application (video consultation, online appointment booking, electronic health record, digital anamnesis). Please consider the statement regardless of whether you have already used this application. *(Responded as a single-choice question)*  **"**I am aware of this digital application.” | | | | | | | | | | | | | | | | | | |
|  | | | | | Yes | | | | | No | | | | | Not specified | | | |
| Video consultations | | | | |  | | | | |  | | | | |  | | | |
| Online appointment booking | | | | |  | | | | |  | | | | |  | | | |
| Electronic health records | | | | |  | | | | |  | | | | |  | | | |
| Digital anamnesis | | | | |  | | | | |  | | | | |  | | | |
| Please indicate your agreement with the following statements for each digital application (video consultation, online appointment booking, electronic health record, digital anamnesis). *(Responded as a single-choice question)*  “The digital application is being offered in my family practice.” | | | | | | | | | | | | | | | | | | |
|  | Yes | | | | | | | No | | | I don´t know | | | | | Not specified | | |
| Video consultations |  | | | | | | |  | | |  | | | | |  | | |
| Online appointment booking |  | | | | | | |  | | |  | | | | |  | | |
| Electronic health records |  | | | | | | |  | | |  | | | | |  | | |
| Digital anamnesis |  | | | | | | |  | | |  | | | | |  | | |
| Please indicate your agreement with the following statements for each digital application (video consultation, online appointment booking, electronic health record, digital anamnesis). *(Responded as a single-choice question)*  “I use this digital application in my family practice."” | | | | | | | | | | | | | | | | | | |
|  | Yes | | | | | | | No | | | The digital application is not offered in my family practice. | | | | | Not specified | | |
| Video consultations |  | | | | | | |  | | |  | | | | |  | | |
| Online appointment booking |  | | | | | | |  | | |  | | | | |  | | |
| Electronic health records |  | | | | | | |  | | |  | | | | |  | | |
| Digital anamnesis |  | | | | | | |  | | |  | | | | |  | | |
| *Next page* | | | | | | | | | | | | | | | | | | |
| **Interest in the use and relevance of digital applications when selecting a family practice**  To what extent do you agree with the following statement? *(Responded as a single-choice question)*  “The extent to which digital applications are used in the family practice is a essential criterion when choosing my family practice."   1. Absolutely disagree 2. Disagree 3. Neither/ Nor 4. Agree 5. Absolutely agree 6. I don´t know   To what extent do you agree with the following statement? Please evaluate the statement separately for the video consultation, online appointment booking, electronic health record and digital anamnesis. *(Responded as a single-choice question)*  “If my family practice does not offer the respective digital application, I would change family practice.” | | | | | | | | | | | | | | | | | | |
|  | | Absolutely disagree | | | Disagree | | Neither/ Nor | | | Agree | | | Absolutely agree | | My family practice already offers the digital application | | | I don´t know |
| Video consultations | |  | | |  | |  | | |  | | |  | |  | | |  |
| Online appointment booking | |  | | |  | |  | | |  | | |  | |  | | |  |
| Electronic health records | |  | | |  | |  | | |  | | |  | |  | | |  |
| Digital anamnesis | |  | | |  | |  | | |  | | |  | |  | | |  |
|  | | | | | | | | | | | | | | | | | | |
| *Next page* | | | | | | | | | | | | | | | | | | |
| **Acceptance of digital applications in the family practice**  Below you will be asked about your intention to use a specific digital application in your family practice. We ask you to rate the following statements for the application presented below. In this way, we want to get to know your previous experience as a user or your expectations as a current non-user toward this digital application. *(Responded as a single-choice question)*  [Participants are randomly assigned to a digital application. To ensure comparability across different digital applications, items are only adapted with the name of the digital applications. Additionally, Participants are shown again descriptions of the digital application assigned to them.]  Please use the following scale to indicate the extent to which you agree with the following statements regarding [video consultation/ online appointment booking/ electronic health record/ digital anamnesis] in a family practice: *(Responded as a single-choice question)* | | | | | | | | | | | | | | | | | | |
|  | | | Strongly disagree | Disagree | | Slightly Disagree | | | Neutral | | | Slightly Agree | | Agree | | | Strongly Agree | |
| I intend to continue using or trying out [video consultations/ online appointment booking/ electronic health records/ digital anamnesis]. | | |  |  | |  | | |  | | |  | |  | | |  | |
| I expect/feel that [video consultations/ online appointment booking/ electronic health records/ digital anamnesis] is easy to use. | | |  |  | |  | | |  | | |  | |  | | |  | |
| I expect / feel that [video consultations/ online appointment booking/ electronic health records/ digital anamnesis] is vulnerable. | | |  |  | |  | | |  | | |  | |  | | |  | |
| I plan to continue or start using [video consultations/ online appointment booking/ electronic health records/ digital anamnesis] on a regular basis. | | |  |  | |  | | |  | | |  | |  | | |  | |
| I expect/feel concerns about privacy issues when using [video consultations/ online appointment booking/ electronic health records/ digital anamnesis]. | | |  |  | |  | | |  | | |  | |  | | |  | |
| I expect / feel that I have the necessary knowledge to use [video consultations/ online appointment booking/ electronic health records/ digital anamnesis]. | | |  |  | |  | | |  | | |  | |  | | |  | |
| I will always try to use [video consultations/ online appointment booking/ electronic health records/ digital anamnesis] in my everyday health care or I would like to integrate [video consultations/ online appointment booking/ electronic health records/ digital anamnesis] into my everyday health care. | | |  |  | |  | | |  | | |  | |  | | |  | |
| I expect/feel that people who are important to me think I should use [video consultations/ online appointment booking/ electronic health records/ digital anamnesis]. | | |  |  | |  | | |  | | |  | |  | | |  | |
| I expect/feel that it is difficult for me to learn how to use [video consultations/ online appointment booking/ electronic health records/ digital anamnesis]. | | |  |  | |  | | |  | | |  | |  | | |  | |
| I expect / feel that [video consultations/ online appointment booking/ electronic health records/ digital anamnesis] is compatible with other technologies and applications that I use. | | |  |  | |  | | |  | | |  | |  | | |  | |
| I expect/feel that it is easy for me to become skillful at using [video consultations/ online appointment booking/ electronic health records/ digital anamnesis]. | | |  |  | |  | | |  | | |  | |  | | |  | |
| I expect/feel that people who influence my behavior think that I should use the [video consultations/ online appointment booking/ electronic health records/ digital anamnesis]. | | |  |  | |  | | |  | | |  | |  | | |  | |
| I expect / feel that I have the necessary resources to use [video consultations/ online appointment booking/ electronic health records/ digital anamnesis]. | | |  |  | |  | | |  | | |  | |  | | |  | |
| I expect / feel that my health information is protected when using [video consultations/ online appointment booking/ electronic health records/ digital anamnesis]. | | |  |  | |  | | |  | | |  | |  | | |  | |
| I expect/feel that using [video consultations/ online appointment booking/ electronic health records/ digital anamnesis] will help me to achieve things faster. | | |  |  | |  | | |  | | |  | |  | | |  | |
| I expect / feel that [video consultations/ online appointment booking/ electronic health records/ digital anamnesis] is useful. | | |  |  | |  | | |  | | |  | |  | | |  | |
| I expect/feel that my interaction with [video consultations/ online appointment booking/ electronic health records/ digital anamnesis] is clear and understandable. | | |  |  | |  | | |  | | |  | |  | | |  | |
| I expect/feel that people whose opinion I value want me to use [video consultations/ online appointment booking/ electronic health records/ digital anamnesis]. | | |  |  | |  | | |  | | |  | |  | | |  | |
| I expect/feel that using [video consultations/ online appointment booking/ electronic health records/ digital anamnesis] will increase my productivity. | | |  |  | |  | | |  | | |  | |  | | |  | |
| The use of [video consultations/ online appointment booking/ electronic health records/ digital anamnesis] in my family practice is or would be optional and voluntary. | | |  |  | |  | | |  | | |  | |  | | |  | |
|  | | | | | | | | | | | | | | | | | | |
| *Next page* | | | | | | | | | | | | | | | | | | |
| Thank you for participating in our study. We would be delighted if you would distribute the survey further. Please use the following link: https://limesurvey.uni-wh.de/DigitaleHausarztpraxis  **References:**  Abd-Alrazaq, Alaa; Bewick, Bridgette M.; Farragher, Tracey; Gardner, Peter (2019): Factors Affecting Patients’ Use of Electronic Personal Health Records in England: Cross-Sectional Study. In: J Med Internet Res 21 (7), e12373. DOI: 10.2196/12373.  Bundesministerium für Gesundheit (2024): Die elektronische Patientenakte (ePA). Online verfügbar unter https://gesund.bund.de/die-elektronische-patientenakte, zuletzt aktualisiert am 27.02.2024, zuletzt geprüft am 27.02.2024.  Harborth, David; Pape, Sebastian (2018): German Translation of the Unified Theory of Acceptance and Use of Technology 2 (UTAUT2) Questionnaire. In: SSRN Electronic Journal. DOI: 10.2139/ssrn.3147708.  Kassenärztliche Bundesvereinigung (KBV) (2024): Videosprechstunde: telemedizinisch gestützte Betreuung von Patienten. Kassenärztliche Bundesvereinigung (KBV). Online verfügbar unter https://www.kbv.de/html/videosprechstunde.php, zuletzt aktualisiert am 27.02.2024, zuletzt geprüft am 27.02.2024.  Spohn, Lucas (2024): Digitale Anamnese – Was lässt sich in acht Minuten Konsultation erreichen? Online verfügbar unter https://www.der-niedergelassene-arzt.de/praxis/news-details/praxisalltag/digitale-anamnese-was-laesst-sich-in-acht-minuten-konsultation-erreichen, zuletzt aktualisiert am 09.02.2024, zuletzt geprüft am 27.02.2024.  Venkatesh, Viswanath; Morris, Michael G.; Davis, Gordon B.; Davis, Fred D. (2003): User Acceptance of Information Technology: Toward a Unified View. In: MIS Quarterly 27 (3), S. 425–478. DOI: 10.2307/30036540.  Whetstone, Melinda; Goldsmith, Ronald (2009): Factors influencing intention to use personal health records. In: International Journal of Pharmaceutical and Healthcare Marketing 3, S. 8–25. DOI: 10.1108/17506120910948485.  Zhao, Peng; Yoo, Illhoi; Lavoie, Jaie; Lavoie, Beau James; Simoes, Eduardo (2017): Web-Based Medical Appointment Systems: A Systematic Review. In: J Med Internet Res 19 (4), e134. DOI: 10.2196/jmir.6747. | | | | | | | | | | | | | | | | | | |
