# Supplementary material for: Patient Adoption of Digital Use Cases in Family Medicine and a Nuanced Implementation Approach for Family Doctors: Quantitative Web-Based Survey Study
Source: JMIR Form Res. 2025 Mar 5;9:e58867. doi: 10.2196/58867 (PMC11923474; doi:10.2196/58867)
Supplement: Multimedia Appendix 3 [file formative_v9i1e58867_app3.docx]

**Multimedia Appendix 3.** CHERRIES (Checklist for Reporting Results of Internet E-Surveys).

**Source: Eysenbach G. (2004). Improving the quality of Web surveys: the Checklist for Reporting Results of Internet E-Surveys (CHERRIES). Journal of medical Internet research, 6(3), e34. https://doi.org/10.2196/jmir.6.3.e34**

| **Item Category** | **Checklist Item** | **Patient Adoption of Digital Use Cases in Family Medicine and a Nuanced Implementation Approach for Family Doctors: Quantitative Web-Based Survey Study** |
| --- | --- | --- |
| **Design** | Describe survey design | The survey compromises five sections: (i) sociodemographic data (gender, age, region of residence, size of residence city, type of insurance, self-assed digital literacy); (ii) previous experience with video consultations, online-appointment systems, electronic medical records and digital anamneses in family practices (awareness regarding digital use case, availability in respective family practice, usage behavior); (iii) relevance of digital use cases when choosing a family practice (digital offering as decision criteria of patients in the selection of family practices; willingness to change general practitioner if selected digital services are not offered); (iv) technology acceptance according to adapted UTAUT model (video consultation or online-appointment systems or digital anamnesis or electronic medical records); (v) personality traits according to Big-Five Model (Openness, Conscientiousness, Extraversion, Agreeableness, Neuroticism).  The scope of this paper only includes sections i-iv, section v will be discussed in a separate research paper. In developing the questionnaire, we leveraged single-choice questions as well as Likert-format questions (e.g., for the self-assessment of digital literacy via rating from 1-10, the relevance of digital use cases when choosing a family practice via “Absolutely disagree” or “Disagree” or “Neither/Nor” or “Agree” or “Absolutely agree”. “Agree” or “Absolutely agree” were interpreted as consent).  Section iv and v leverage existing questionnaires: Section iv uses a questionnaire developed by Abd-Alrazaq et al. [1] due to its demonstrated validity and reliability in a primary care setting. This questionnaire was adapted to the other use cases and translated into German language based on existing validated translations of the UTAUT construct [2]. In doing so, we kept the original 7-point Likert scale. Section v leverages the BFI-K by Rammstedt & John [3] with no further adaptation (thus we kept the original 5-Point Likert scale).  The goal of our convenience sampling approach was to recruit a large cross-section sample size of adults (over 18 years) living in Germany. Eventually, 2156 participants finished our survey of which 1880 entries were incorporated for data analysis. Appropriate data cleaning mechanisms included among others removal of duplicate entries, removal of entries with no reasonable answer behavior (straight lining) and exclusion of fasted 5% answers of study. To enhance the completion rate, we forced responses for all sections – however, we provided the answer option ”Not specified” respective “I don´t know” for sections i to iii.  Potential participants were shown an introductory page before the start of the survey. On this introductory page, research intent and objectives, context of the survey, survey length, study responsibility, IRB approval and target sample were clarified. Contacts of the researchers were included in case of any further questions. On the next page, participants were asked to give informed consent to our privacy policy which further elaborated on reasons, responsible researchers and measures of data processing as well as the rights of participants of the survey involved. Contact details of appropriate data security authorities have been provided. Potential participants were not able to continue with the survey if they did not provide consent to the data privacy policy.  No personal information was collected. Sociodemographic data was anonymized according to k>=5 anonymity. All gathered information was stored on servers of the Witten/Herdecke University. |
| **IRB (Institutional Review Board) approval and informed consent process** | IRB approval | Our study was accepted by the Ethics Committee of Witten/Herdecke University prior to start (Nr. S-245/2022). In developing the data privacy policy, we reviewed the incorporation of GDPR principles together with the data protection officer of the Witten/Herdecke University. |
|  | Informed consent | Potential participants were shown an introductory page before the start of the survey. On this introductory page, research intent and objectives, survey length, study responsibility, IRB approval and target sample were clarified. Contact possibilities were included in case of any further questions. On the next page, participants were asked to give informed consent to our privacy policy which further elaborated on reasons, responsibilities and measures of data processing as well as the rights of participants involved. Contact details of appropriate data security authorities have been provided. Potential participants were not able to continue with the survey if they did not provide consent to the data privacy policy. |
|  | Data protection | No personal information was collected. Sociodemographic data was anonymized according to k>=5 anonymity. All gathered information was stored on servers of the Witten/Herdecke University. |
| **Development and pre-testing** | Development and testing | With respect to our research focus, we decided to conduct a rapid literature review, aligned to PRISMA-RR as well as recommendations given by Tricco et al. [4] and King et al. [5]. As PRISMA-RR is still under development, we adapted PRISMA guidelines (especially PRIMSA-ScR) to the constraints of our project. In our literature research, we screened three different databases (PubMed, Psychinfo and Scopus) with a search strategy based on the connection of three building blocks with the Boolean “OR” operator: 1) technology acceptance models and 2) patient views and 3) the respective digital use case. All building blocks consist of different wordings / underlying concepts of the overarching name of the building block. Search results were limited to being in German or English and being published between 2018 and 2023. In addition, relevant research papers from e.g., citation searching were included. Items for sections i-iii were developed based on existing research papers assessing attitudes towards digital technologies [6] as well as based on a joint decision of different researchers with a background in the digitalization of primary care.  For section iv assessing technology acceptance: Building on insights on patient acceptance of the digital applications in scope, we leveraged an existing questionnaire assessing the acceptance of electronic medical records in primary care in the UK based on the UTAUT model [1] and adopted it to the other use cases. We chose to use an existing questionnaire of Abd-Alrazaq et al. [1] due to its demonstrated validity and reliability in a primary care setting (e.g., average Cronbach´s Alpha of .95, average Average Variance Contracted (AVE) of .95; and average Composite Reliability (CR) values of .87) as well as its comprehensive justification of the appropriate technology acceptance model for the context of the primary care setting. The questionnaire extends the UTAUT model based on Venkatesh et al. [7] with perceived privacy & security items [8] which emerged also in our literature review as an additional element to understand technology acceptance in healthcare settings. Items which were dropped in the original model of Abd-Alrazaq et al. ([1] due to lower factor loading were excluded from our questionnaire.  For our research, we replicated this validated research instrument in the healthcare setting to the other three use cases. To ensure comparability along items of different use cases, we replaced the wording for the “patient portal” respective “electronic medical record” of the original questionnaire with the name of the other use cases. The questionnaire was then translated into German language based on existing validated translations of the UTAUT construct [2].  The questionnaire was pre-tested for technical functionality, comprehensibility and usability with 21 participants from the sample target group and feedback was gathered via written input as well as orally via phone calls and voice memos. Minor adjustments regarding answer options, order of sections as well as the wording of selected items were made. |
| **Recruitment process and description of the sample having access to the questionnaire** | Open survey versus closed survey | We used an open-access mode for our survey implying that everyone with the link could access the survey. |
|  | Contact mode | Except for the personal contact of the direct network of the researcher and the hand-out of physical flyers advertising the survey (flyers had a QR-Code for access printed on them), initial contact was always made via the internet. |
|  | Advertising the survey | The researchers used various recruitment channels to gather a sample size of adults visiting primary doctors in Germany as large and diverse as possible. Since the only limiting factor was reaching legal age, our sample frame was very broad. Therefore, the main recruitment channels included:   - the Social Sciences’ Panel (a convenience pool project of the Institute for Communication Science and Media Research (LMU Munich) and the German Communication Association (DGPuK)), - social media platforms such as e.g., LinkedIn, Instagram and Facebook: Direct contact of researcher´s personal network as well as direct contact with members of various LinkedIn groups expressing interest in digitalization and/or healthcare - online (research) forums such as e.g., Reddit, Psychologie Heute, studies-online, Wiwi-Treff and SurveyCircle - the distribution of physical flyers in waiting rooms of family practices and other facilities   The wording of the announcement was adopted appropriately according to the channel:   - For Social Sciences’ Panel:   - Reference line of e-mail: [SoSci-Studie] Sollte Ihre Hausarztpraxis überhaupt digitaler werden? Und falls ja, was ist Ihnen dabei wichtig?   - Text: Liebe Panelteilnehmerin, lieber Panelteilnehmer,   - am Lehrstuhl für Gesundheitsinformatik der Universität Witten/Herdecke untersuche ich in meiner Doktorarbeit, was volljährige Patient:innen von digitalen Anwendungen in Hausarztpraxen erwarten. Um die Forschung in Deutschland dabei maßgeblich voranzubringen, brauche ich Ihre Unterstützung: Teilen Sie Ihre Erwartungen als Patient:in in unserer ca. 10-minütigen und anonymen Umfrage und gestalten Sie so die Zukunft der hausärztlichen Versorgung aktiv mit.   - Hier geht es zum Fragebogen: https://www.soscipanel.de/?survey=NWIAB0   - Der Fragebogen ist mit dem Ziel der Barrierefreiheit konzipiert worden, nichtsdestotrotz könnten Einschränkungen für sehbehinderte Personen auftreten.   - Vielen Dank für Ihren Beitrag zu meiner Doktorarbeit und zur wissenschaftlichen Forschung! - Direct contact via social media platforms:   - Hallo XYZ, Ich habe auf LinkedIn gesehen, dass Sie zu Digitalisierungsthemen im Gesundheitswesen aktiv sind. Das finde ich sehr spannend, da ich selbst ich in meiner Doktorarbeit untersuche, was Patient:innen von digitalen Anwendungen in Hausarztpraxen erwarten.   - Um die Forschung in Deutschland dabei maßgeblich voranzubringen, brauche ich Ihre Unterstützung: Teilen Sie Ihre Erwartungen in unserer ca. 10-minütigen und anonymen Umfrage und gestalten Sie so die Zukunft der Digitalisierung in Deutschland aktiv mit.   - https://limesurvey.uni-wh.de/DigitaleHausarztpraxis   - Vielen Dank für Ihren Beitrag zu meiner Doktorarbeit und zur wissenschaftlichen Forschung in Deutschland,   - Julian Beerbaum - Posting in research channels:   - Umfrage: Sollte Deine Hausarztpraxis überhaupt digitaler werden? Und falls ja, was ist Dir dabei wichtig?   - Hallo zusammen,   - am Lehrstuhl für Gesundheitsinformatik der Universität Witten/Herdecke untersuche ich in meiner Doktorarbeit, was Patient:innen von digitalen Anwendungen in Hausarztpraxen erwarten.   - Um die Forschung in Deutschland dabei maßgeblich voranzubringen, brauche ich Eure Unterstützung: Teilt Eure Erwartungen in unserer ca. 10-minütigen und anonymen Umfrage und gestaltet so die Zukunft der hausärztlichen Versorgung aktiv mit. Die Umfrage ist noch bis Anfang Dezember online.   - https://limesurvey.uni-wh.de/DigitaleHausarztpraxis   - Vielen Beitrag für Euren Beitrag zur wissenschaftlichen Forschung in Deutschland! - Physical flyers:   - Sehr geehrte(r) Studieninteressierte(r),   - sollte Ihre Hausarztpraxis überhaupt digitaler werden? Und falls ja, was ist Ihnen dabei wichtig?  Am Lehrstuhl für Gesundheitsinformatik der Universität Witten/Herdecke untersuche ich in meiner Doktorarbeit, was Patient:innen von digitalen Anwendungen in Hausarztpraxen erwarten.  Um die Forschung in Deutschland dabei maßgeblich voranzubringen, brauche ich Ihre Unterstützung: Teilen Sie Ihre Erwartungen in unserer ca. 10-minütigen und anonymen Umfrage und gestalten Sie so die Zukunft der hausärztlichen Versorgung aktiv mit.   - Vielen Dank für Ihren Beitrag zu meiner Doktorarbeit und zur wissenschaftlichen Forschung,   - Julian Beerbaum |
| **Survey administration** | Web/E-Mail | The researchers used the web-based survey tool LimeSurvey to build the survey, to gather responses automatically and to create the right export datasheets for data analysis. Thus, the questionnaire was only posted on the web and no manual addition of responses from other sources e.g., physical questionnaires has been conducted. We are aware that the use of only a web-based survey tool might bias the sample size towards having a higher digital literacy. We tried to counter this effect by cooperating with family practices to hand out physical questionnaires in the waiting rooms, however responses to the ask of even only distributing small A6 flyers were limited (only three responses out of over 500 contacted family practices). |
|  | Context | The only automatic exclusion factor for respondents of our survey was not reaching legal age (having visited a family doctor in the last twelve months as an automatic exclusion factor has been dropped after running the survey for several weeks due to higher-than-expected drop-out rates). Therefore, to gather large and diverse sample size as possible, various recruitment channels have been leveraged with different themes. Nevertheless, due to the voluntary nature of our survey, we are likely to have a bias towards people who are interested in digitalization and/or primary care. |
|  | Mandatory/voluntary | Filling out the survey was voluntary. |
|  | Incentives | We did not provide any incentives to fill out the survey however offered individuals to send them our final research papers. |
|  | Time/Date | The survey was conducted on LimeSurvey between the 30th of May 2023 and the 22nd of November 2023. |
|  | Randomization of items or questionnaires | In total, our survey encompassed five sections. Regarding the order of sections, we saw no risk of biased answering behavior due to the type of information requested (e.g., sociodemographic information or experience with digital use cases in scope). For sections iv and v, we leveraged existing questionnaires and kept the same order of items within this section to keep the reliability and viability of the original surveys. |
|  | Adaptive questioning | After answering closed-sociodemographic questions, participants were shown brief descriptions of all the digital technologies in the scope of this paper to minimize any misunderstanding bias. Participants were then randomly assigned to one of the digital use cases for which they answered the technology acceptance items on a Likert Scale. The number of use cases per participant was later reduced from two to one use case due to higher-than-expected drop-out rates. This approach was appropriate as the order of questions was not changed. |
|  | Number of items | The number of items per page had to be reduced to use the Social Sciences Panel. Therefore, two separate links had been created - one for the Social Sciences’ Panel and one for all other recruitment channels. Both links have the same questions in the same order and only the average number of items per page differed. In total, the survey encompassed 66 questionnaire items (excluding forwarding on the introductory page and consenting to the data privacy policy on the next page). The number of questions per page was the same for sections i to iii for both links: Section I entails 7 single-choice items and one rating item across two pages (two items for page 3 and 6 items for page 4). Section ii consists of 12 single-choice items on one page and section iii compromises five Likert-scale questions on one page.  Sections iv and v differed between the link for the Social Sciences’ Panel and the link for all other recruitment channels. While the Social Sciences’ Panel distributes the 20 technology acceptance Likert-scale items across three pages (7 items per page for two pages, 6 items per page for one page), the link used for all other recruiting channels contains all 20 items on one page. The same is true for the personality assessment as the Social Sciences’ Panel spreads out the 21 Likert-scale questions across three pages (7 items per page) while the link for all other channels only uses one page. To conclude, the average number of items per page for the whole survey for the Social Sciences Link was 6.6 while the average number of items per page for the other link was 11. |
|  | Number of screens (pages) | As stated above, two separate links have been created - one for the Social Sciences’ Panel and one for all other recruitment channels. Both links have the same questions in the same order and only the average number of items per page differed. In total, the survey encompassed 66 questionnaire items (excluding forwarding on the introductory page and consenting to the data privacy policy on the next page). The link for the Social Sciences’ Panel used 10 pages while the link for all other recruitment channels leveraged 6 pages. |
|  | Completeness check | To enhance the completion rate, we forced responses for all sections – however, we provided the answer option ”Not specified” respective “I don´t know” for sections i to iii. |
|  | Review step | To mitigate any social desirability bias, we did not add a back button or a review step. |
| **Response rates** | Unique site visitor | 2.714 potential participants accessed the survey. We used cookies to prevent double data entry. Additionally, we excluded entries which showed the same IP address as well as the same sociodemographic characteristics as a previous entry (see section "Preventing multiple entries from the same individual"). |
|  | View rate | Not applicable as a variety of recruitment channels have been used. |
|  | Participation rate | 2.714 potential participants accessed the survey of which 2.596 potential participants read the introductory page and gave consent to the data privacy policy. Thus, the participation rate is 95.6%. |
|  | Completion rate | 2.596 potential participants read the introductory page and gave consent to the data privacy policy. Of these participants, 2.156 completed the last survey page. Thus, the completion rate is 83.1%. |
| **Preventing multiple entries from the same individual** | Cookies used | Cookies were used to prevent repeated data entries of the same participant. Participants who already filled out the survey were shown an error message when accessing the survey again. |
|  | IP check | Respondents had their time stamps and anonymized IP addresses logged. Even though we used cookies to prevent repeated access, the researchers conducted an additional IP check to avoid repeat participation (e.g., the participant deleted his cookies after his first data entry). Therefore, we highlighted complete data entries that showed the same IP address as well as all the same demographic data. Of those marked 52 entries, we kept the first entry for data analysis, thus finally excluding the later 26 entries. |
|  | Log file analysis | We did not take any measures to conduct a log file analysis. |
|  | Registration | The survey was conducted in an open-access mode. |
| **Analysis** | Handling of incomplete questionnaires | We only included participants of our survey who completed the whole survey (including section v which is not in the scope of this paper). We believe this approach is adequate because we hypothesize that participants who dropped out of the survey might also be more likely to fill out the overall survey with less diligence. Thus, we believe that by only including participants who completed the whole survey, we enhance the reliability and viability of our research. |
|  | Questionnaires submitted with atypical timestamp | The following data cleaning measures were conducted in hierarchical order, meaning that the new data basis already incorporates the previous data cleaning steps. To ensure the quality of our survey, we excluded the fastest 5% of complete with no duplicate data entries. This cut-off point seemed appropriate as trials of completing the survey as fast as possible while reading all the information took longer than the cut-off time. Thus, we do not expect diligent answering behavior of the top 5% fastest survey respondents. This meant that we excluded a further 104 responses. Furthermore, we controlled for appropriate answering behavior with two means. First, we excluded 23 responses that showed straight lining in either section I, iv or v. Second, we excluded 56 responses that answered a first control question incorrectly and 13 participants that answered a second control question incorrectly. Additionally, we removed 47 entries of "not specified" in the sociodemographic section to further allow for meaningful data analysis of the impact of sociodemographic characteristics on technology acceptance. Lastly, we eliminated 8 responses for at least one item that violated k>=5 anonymity per research model (e.g., for video consultation) in line with our data privacy policy. In total, we included 1880 entries for data analysis. |
|  | Statistical correction | We did not apply any statistical correction. |

**References**

1. Abd-Alrazaq A, Bewick BM, Farragher T, Gardner P. Factors Affecting Patients’ Use of Electronic Personal Health Records in England: Cross-Sectional Study. J Med Internet Res 2019;21(7):e12373. doi:10.2196/12373
2. Harborth D, Pape S. German Translation of the Unified Theory of Acceptance and Use of Technology 2 (UTAUT2) Questionnaire. SSRN Electronic Journal 2018. doi:10.2139/ssrn.3147708
3. Rammstedt, Beatrice & John, Oliver. Kurzversion des Big Five Inventory (BFI-K) [Short version of the Big Five Inventory (BFI-K)]. In *Diagnostica*. 51 2015. 195-206. DOI: 10.1026/0012-1924.51.4.195.
4. Tricco AC, Zarin W, Antony J, Hutton B, Moher D, Sherifali D, Straus SE. An international survey and modified Delphi approach revealed numerous rapid review methods. J Clin Epidemiol 2016;70:61-67. doi:10.1016/j.jclinepi.2015.08.012
5. King VJ, Stevens A, Nussbaumer-Streit B, Kamel C, Garritty C. Paper 2: Performing rapid reviews. Systematic Reviews 2022;11(1):151. doi:10.1186/s13643-022-02011-5
6. Dahlhausen, F., Zinner, M., Bieske, L., Ehlers, J. P., Boehme, P., & Fehring, L. Physicians' Attitudes Toward Prescribable mHealth Apps and Implications for Adoption in Germany: Mixed Methods Study. JMIR mHealth and uHealth 2021, 9(11), e33012. https://doi.org/10.2196/33012
7. Venkatesh V, Morris MG, Davis GB, Davis FD. User Acceptance of Information Technology: Toward a Unified View. MIS Quarterly 2003;27(3):425-478. doi:10.2307/30036540
8. Whetstone M, Goldsmith R. Factors influencing intention to use personal health records. International Journal of Pharmaceutical and Healthcare Marketing 2009;3:8-25. doi:10.1108/17506120910948485
