# Supplementary material for: Patient Adoption of Digital Use Cases in Family Medicine and a Nuanced Implementation Approach for Family Doctors: Quantitative Web-Based Survey Study
Source: JMIR Form Res. 2025 Mar 5;9:e58867. doi: 10.2196/58867 (PMC11923474; doi:10.2196/58867)
Supplement: Multimedia Appendix 4 [file formative_v9i1e58867_app4.docx]

**Multimedia Appendix 4.** Overview of regression coefficients for the regression models predicting the intention to use digital use cases.

1. **Receiving medical consultations via video (n=494)**

| **Model** | **Variable** | **B** | **SE B** | ***β*** | ***P* value** | **95% CI** |
| --- | --- | --- | --- | --- | --- | --- |
| **1** | (Constant) | 0.960 | 0.641 |  | .14 | -0.30 ; 2.22 |
|  | Frequency of doctor visits: 1 visit vs. no doctor visit in last 12 months | 0.141 | 0.356 | .032 | .69 | -0.56 ; 0.84 |
|  | Frequency of doctor visits: 2-3 visits vs. no doctor visit in last 12 months | 0.487 | 0.337 | .134 | .15 | -0.18 ; 1.15 |
|  | Frequency of doctor visits: >3 visits vs. no doctor visit in last 12 months | 0.505 | 0.341 | .137 | .14 | -0.16 ; 1.17 |
|  | Gender: female vs. male | 0.321 | 0.159 | .090 | **.**04 | 0.01 ; 0.63 |
|  | Age:31-50 years vs.18-30 years | 0.352 | 0.230 | .087 | .13 | -0.10 ; 0.80 |
|  | Age:51-65 years vs.18-30 years | 0.410 | 0.240 | .107 | .09 | -0.06 ; 0.88 |
|  | Age:>65 years vs.18-30 years | 0.137 | 0.264 | .032 | .60 | -0.38 ; 0.66 |
|  | Size of residence city: 20,001 to 200, 000 vs. <= 20,000 | -0.099 | 0.202 | -.025 | .62 | -0.50 ; 0.30 |
|  | Size of residence city: >200,000 vs. <= 20,000 | -0.206 | 0.199 | -.057 | .30 | -0.60 ; 0.18 |
|  | Region: South vs. East | -0.297 | 0.244 | -.061 | .22 | -0.78 ; 0.18 |
|  | Region: West vs. East | -0.272 | 0.236 | -.061 | .25 | -0.73 ; 0.19 |
|  | Region: North vs. East | -0.095 | 0.201 | -.026 | .64 | -0.49 ; 0.30 |
|  | Type of insurance: Private vs. Statutory | -0.188 | 0.190 | -.044 | .32 | -0.56 ; 0.19 |
|  | Digital literacy | 0.341 | 0.049 | .335 | <.001 | 0.25 ; 0.44 |
| **2** | (Constant) | -2.270 | 0.478 |  | <.001 | -3.21 ; -1.33 |
|  | Frequency of doctor visits: 1 visit vs. no doctor visit in last 12 months | -0.243 | 0.236 | -.055 | .31 | -0.71 ; 0.22 |
|  | Frequency of doctor visits: 2-3 visits vs. no doctor visit in last 12 months | 0.006 | 0.223 | .002 | .98 | -0.43 ; 0.44 |
|  | Frequency of doctor visits: >3 visits vs. no doctor visit in last 12 months | -0.101 | 0.226 | -.027 | .65 | -0.55 ; 0.34 |
|  | Gender: female vs. male | 0.142 | 0.106 | .040 | .18 | -0.06 ; 0.35 |
|  | Age:31-50 years vs.18-30 years | 0.378 | 0.152 | .094 | .01 | 0.08 ; 0.68 |
|  | Age:51-65 years vs.18-30 years | 0.653 | 0.159 | .171 | .00 | 0.34 ; 0.97 |
|  | Age:>65 years vs.18-30 years | 0.496 | 0.178 | .117 | .01 | 0.15 ; 0.85 |
|  | Size of residence city: 20,001 to 200, 000 vs. <= 20,000 | -0.036 | 0.133 | -.009 | .79 | -0.30 ; 0.23 |
|  | Size of residence city: >200,000 vs. <= 20,000 | -0.115 | 0.131 | -.032 | .38 | -0.37 ; 0.14 |
|  | Region: South vs. East | -0.190 | 0.161 | -.039 | .24 | -0.51 ; 0.13 |
|  | Region: West vs. East | -0.145 | 0.156 | -.033 | .35 | -0.45 ; 0.16 |
|  | Region: North vs. East | -0.047 | 0.132 | -.013 | .72 | -0.31 ; 0.21 |
|  | Type of insurance: Private vs. Statutory | -0.069 | 0.125 | -.016 | .58 | -0.32 ; 0.18 |
|  | Digital literacy | 0.050 | 0.036 | .049 | .16 | -0.02 ; 0.12 |
|  | Performance Expectancy | 0.603 | 0.049 | .527 | . <.001 | 0.51 ; 0.70 |
|  | Effort Expectancy | 0.236 | 0.084 | .135 | .005 | 0.07 ; 0.40 |
|  | Social Influence | 0.227 | 0.036 | .206 | . <.001 | 0.16 ; 0.30 |
|  | Facilitating Condition | 0.063 | 0.069 | .039 | .36 | -0.07 ; 0.20 |
|  | Perceived Privacy and Security | 0.068 | 0.042 | .053 | .10 | -0.01 ; 0.15 |

**Note:** Table displays the regression coefficients for our regression model. SE = standard error for regression coefficient B; 95% CI presents upper and lower bound for the 95% confidence interval for B.

1. **Scheduling doctor appointments online (n=542)**

| **Model** | **Variable** | **B** | **SE B** | ***β*** | ***P* value** | **95% CI** |
| --- | --- | --- | --- | --- | --- | --- |
| **1** | (Constant) | 2.390 | 0.478 |  | . <.001 | 1.45 ; 3.33 |
|  | Frequency of doctor visits: 1 visit vs. no doctor visit in last 12 months | 0.783 | 0.285 | .213 | .01 | 0.22 ; 1.34 |
|  | Frequency of doctor visits: 2-3 visits vs. no doctor visit in last 12 months | 0.698 | 0.267 | .233 | .01 | 0.17 ; 1.22 |
|  | Frequency of doctor visits: >3 visits vs. no doctor visit in last 12 months | 0.690 | 0.270 | .224 | .01 | 0.16 ; 1.22 |
|  | Gender: female vs. male | 0.247 | 0.122 | .084 | .04 | 0.01 ; 0.49 |
|  | Age:31-50 years vs.18-30 years | -0.036 | 0.180 | -.011 | .84 | -0.39 ; 0.32 |
|  | Age:51-65 years vs.18-30 years | -0.071 | 0.182 | -.023 | .69 | -0.43 ; 0.29 |
|  | Age:>65 years vs.18-30 years | -0.294 | 0.208 | -.083 | .16 | -0.7 ; 0.11 |
|  | Size of residence city: 20,001 to 200, 000 vs. <= 20,000 | 0.072 | 0.162 | .022 | .66 | -0.25 ; 0.39 |
|  | Size of residence city: >200,000 vs. <= 20,000 | 0.094 | 0.151 | .032 | .53 | -0.2 ; 0.39 |
|  | Region: South vs. East | 0.018 | 0.191 | .005 | .93 | -0.36 ; 0.39 |
|  | Region: West vs. East | -0.127 | 0.182 | -.035 | .48 | -0.48 ; 0.23 |
|  | Region: North vs. East | 0.088 | 0.160 | .029 | .58 | -0.23 ; 0.4 |
|  | Type of insurance: Private vs. Statutory | -0.033 | 0.143 | -.010 | .81 | -0.31 ; 0.25 |
|  | Digital literacy | 0.322 | 0.033 | .408 | . <.001 | 0.26 ; 0.39 |
| **2** | (Constant) | -2.125 | 0.441 |  | .00 | -2.99 ; -1.26 |
|  | Frequency of doctor visits: 1 visit vs. no doctor visit in last 12 months | 0.207 | 0.197 | .056 | .29 | -0.18 ; 0.59 |
|  | Frequency of doctor visits: 2-3 visits vs. no doctor visit in last 12 months | 0.069 | 0.185 | .023 | .71 | -0.3 ; 0.43 |
|  | Frequency of doctor visits: >3 visits vs. no doctor visit in last 12 months | 0.055 | 0.187 | .018 | .77 | -0.31 ; 0.42 |
|  | Gender: female vs. male | 0.188 | 0.083 | .064 | .02 | 0.02 ; 0.35 |
|  | Age:31-50 years vs.18-30 years | 0.172 | 0.124 | .052 | .17 | -0.07 ; 0.42 |
|  | Age:51-65 years vs.18-30 years | 0.264 | 0.127 | .085 | .04 | 0.01 ; 0.51 |
|  | Age:>65 years vs.18-30 years | 0.383 | 0.145 | .108 | .01 | 0.1 ; 0.67 |
|  | Size of residence city: 20,001 to 200, 000 vs. <= 20,000 | 0.002 | 0.111 | .001 | .99 | -0.22 ; 0.22 |
|  | Size of residence city: >200,000 vs. <= 20,000 | 0.066 | 0.104 | .023 | .52 | -0.14 ; 0.27 |
|  | Region: South vs. East | 0.004 | 0.131 | .001 | .97 | -0.25 ; 0.26 |
|  | Region: West vs. East | -0.080 | 0.125 | -.022 | .52 | -0.32 ; 0.17 |
|  | Region: North vs. East | 0.076 | 0.110 | .025 | .49 | -0.14 ; 0.29 |
|  | Type of insurance: Private vs. Statutory | -0.167 | 0.098 | -.048 | .09 | -0.36 ; 0.03 |
|  | Digital literacy | 0.088 | 0.025 | .112 | . <.001 | 0.04 ; 0.14 |
|  | Performance Expectancy | 0.566 | 0.043 | .513 | . <.001 | 0.48 ; 0.65 |
|  | Effort Expectancy | 0.391 | 0.075 | .218 | . <.001 | 0.24 ; 0.54 |
|  | Social Influence | 0.046 | 0.025 | .054 | .07 | 0 ; 0.1 |
|  | Facilitating Condition | 0.047 | 0.060 | .028 | .44 | -0.07 ; 0.17 |
|  | Perceived Privacy and Security | 0.117 | 0.037 | .094 | . <.001 | 0.04 ; 0.19 |

**Note:** Table displays the regression coefficients for our regression model. SE = standard error for regression coefficient B; 95% CI presents upper and lower bound for the 95% confidence interval for B.

1. **Storing personal medical information via electronic health records (n=528)**

| **Model** | **Variable** | **B** | **SE B** | ***β*** | ***P* value** | **95% CI** |
| --- | --- | --- | --- | --- | --- | --- |
| **1** | (Constant) | 2.142 | 0.530 |  | . <.001 | 1.10 ; 3.18 |
|  | Frequency of doctor visits: 1 visit vs. no doctor visit in last 12 months | -0.034 | 0.330 | -.008 | .92 | -0.68 ; 0.61 |
|  | Frequency of doctor visits: 2-3 visits vs. no doctor visit in last 12 months | 0.516 | 0.315 | .142 | .10 | -0.10 ; 1.13 |
|  | Frequency of doctor visits: >3 visits vs. no doctor visit in last 12 months | 0.243 | 0.318 | .066 | .45 | -0.38 ; 0.87 |
|  | Gender: female vs. male | -0.356 | 0.149 | -.101 | .02 | -0.65 ; -0.06 |
|  | Age:31-50 years vs.18-30 years | -0.069 | 0.219 | -.017 | .75 | -0.50 ; 0.36 |
|  | Age:51-65 years vs.18-30 years | -0.082 | 0.225 | -.021 | .71 | -0.53 ; 0.36 |
|  | Age:>65 years vs.18-30 years | 0.082 | 0.248 | .020 | .74 | -0.41 ; 0.57 |
|  | Size of residence city: 20,001 to 200, 000 vs. <= 20,000 | 0.214 | 0.201 | .056 | .29 | -0.18 ; 0.61 |
|  | Size of residence city: >200,000 vs. <= 20,000 | 0.080 | 0.189 | .023 | .67 | -0.29 ; 0.45 |
|  | Region: South vs. East | -0.130 | 0.232 | -.028 | .57 | -0.59 ; 0.33 |
|  | Region: West vs. East | -0.141 | 0.222 | -.031 | .53 | -0.58 ; 0.29 |
|  | Region: North vs. East | 0.190 | 0.195 | .052 | .33 | -0.19 ; 0.57 |
|  | Type of insurance: Private vs. Statutory | -0.093 | 0.179 | -.022 | .60 | -0.45 ; 0.26 |
|  | Digital literacy | 0.308 | 0.041 | .329 | . <.001 | 0.23 ; 0.39 |
| **2** | (Constant) | -1.614 | 0.390 |  | . <.001 | -2.38 ; -0.85 |
|  | Frequency of doctor visits: 1 visit vs. no doctor visit in last 12 months | -0.121 | 0.220 | -.029 | .58 | -0.55 ; 0.31 |
|  | Frequency of doctor visits: 2-3 visits vs. no doctor visit in last 12 months | -0.003 | 0.210 | -.001 | .99 | -0.42 ; 0.41 |
|  | Frequency of doctor visits: >3 visits vs. no doctor visit in last 12 months | -0.055 | 0.212 | -.015 | .80 | -0.47 ; 0.36 |
|  | Gender: female vs. male | -0.114 | 0.100 | -.032 | .26 | -0.31 ; 0.08 |
|  | Age:31-50 years vs.18-30 years | 0.046 | 0.146 | .012 | .75 | -0.24 ; 0.33 |
|  | Age:51-65 years vs.18-30 years | 0.244 | 0.151 | .063 | .11 | -0.05 ; 0.54 |
|  | Age:>65 years vs.18-30 years | 0.379 | 0.167 | .091 | .02 | 0.05 ; 0.71 |
|  | Size of residence city: 20,001 to 200, 000 vs. <= 20,000 | 0.012 | 0.134 | .003 | .93 | -0.25 ; 0.28 |
|  | Size of residence city: >200,000 vs. <= 20,000 | -0.108 | 0.126 | -.031 | .39 | -0.35 ; 0.14 |
|  | Region: South vs. East | -0.127 | 0.154 | -.027 | .41 | -0.43 ; 0.18 |
|  | Region: West vs. East | 0.064 | 0.148 | .014 | .67 | -0.23 ; 0.35 |
|  | Region: North vs. East | -0.077 | 0.130 | -.021 | .55 | -0.33 ; 0.18 |
|  | Type of insurance: Private vs. Statutory | -0.193 | 0.119 | -.045 | .11 | -0.43 ; 0.04 |
|  | Digital literacy | 0.060 | 0.030 | .064 | .05 | 0 ; 0.12 |
|  | Performance Expectancy | 0.405 | 0.047 | .348 | . <.001 | 0.31 ; 0.50 |
|  | Effort Expectancy | 0.153 | 0.070 | .096 | .03 | 0.02 ; 0.29 |
|  | Social Influence | 0.108 | 0.033 | .101 | . <.001 | 0.04 ; 0.17 |
|  | Facilitating Condition | 0.299 | 0.061 | .198 | . <.001 | 0.18 ; 0.42 |
|  | Perceived Privacy and Security | 0.284 | 0.040 | .243 | <.001 | 0.21 ; 0.36 |

**Note:** Table displays the regression coefficients for our regression model. SE = standard error for regression coefficient B; 95% CI presents upper and lower bound for the 95% confidence interval for B.

1. **Providing personal information pre-consultation digitally (digital anamnesis) (n=491)**

| **Model** | **Variable** | **B** | **SE B** | ***β*** | ***P* value** | **95% CI** |
| --- | --- | --- | --- | --- | --- | --- |
| **1** | (Constant) | 2.496 | 0.536 |  | .00 | 1.44 ; 3.55 |
|  | Frequency of doctor visits: 1 visit vs. no doctor visit in last 12 months | 0.098 | 0.302 | .025 | .74 | -0.49 ; 0.69 |
|  | Frequency of doctor visits: 2-3 visits vs. no doctor visit in last 12 months | 0.313 | 0.285 | .089 | .27 | -0.25 ; 0.87 |
|  | Frequency of doctor visits: >3 visits vs. no doctor visit in last 12 months | 0.479 | 0.290 | .136 | .10 | -0.09 ; 1.05 |
|  | Gender: female vs. male | -0.125 | 0.153 | -.037 | .41 | -0.43 ; 0.17 |
|  | Age:31-50 years vs.18-30 years | 0.049 | 0.216 | .013 | .82 | -0.38 ; 0.47 |
|  | Age:51-65 years vs.18-30 years | -0.476 | 0.235 | -.125 | .04 | -0.94 ; -0.01 |
|  | Age:>65 years vs.18-30 years | -0.614 | 0.239 | -.158 | .01 | -1.08 ; -0.14 |
|  | Size of residence city: 20,001 to 200, 000 vs. <= 20,000 | 0.025 | 0.197 | .007 | .90 | -0.36 ; 0.41 |
|  | Size of residence city: >200,000 vs. <= 20,000 | -0.018 | 0.192 | -.005 | .92 | -0.4 ; 0.36 |
|  | Region: South vs. East | -0.173 | 0.244 | -.038 | .48 | -0.65 ; 0.31 |
|  | Region: West vs. East | 0.091 | 0.232 | .022 | .69 | -0.36 ; 0.55 |
|  | Region: North vs. East | 0.067 | 0.201 | .020 | .74 | -0.33 ; 0.46 |
|  | Type of insurance: Private vs. Statutory | 0.271 | 0.181 | .067 | .13 | -0.08 ; 0.63 |
|  | Digital literacy | 0.239 | 0.044 | .250 | . <.001 | 0.15 ; 0.33 |
| **2** | (Constant) | -1.616 | 0.405 |  | .00 | -2.41 ; -0.82 |
|  | Frequency of doctor visits: 1 visit vs. no doctor visit in last 12 months | -0.192 | 0.201 | -.048 | .34 | -0.59 ; 0.2 |
|  | Frequency of doctor visits: 2-3 visits vs. no doctor visit in last 12 months | -0.131 | 0.189 | -.037 | .49 | -0.50 ; 0.24 |
|  | Frequency of doctor visits: >3 visits vs. no doctor visit in last 12 months | -0.089 | 0.192 | -.025 | .64 | -0.46 ; 0.29 |
|  | Gender: female vs. male | 0.017 | 0.102 | .005 | .87 | -0.18 ; 0.22 |
|  | Age:31-50 years vs.18-30 years | 0.187 | 0.141 | .050 | .19 | -0.09 ; 0.46 |
|  | Age:51-65 years vs.18-30 years | 0.215 | 0.157 | .057 | .17 | -0.09 ; 0.52 |
|  | Age:>65 years vs.18-30 years | 0.144 | 0.160 | .037 | .37 | -0.17 ; 0.46 |
|  | Size of residence city: 20,001 to 200, 000 vs. <= 20,000 | 0.019 | 0.128 | .005 | .88 | -0.23 ; 0.27 |
|  | Size of residence city: >200,000 vs. <= 20,000 | 0.044 | 0.126 | .013 | .73 | -0.2 ; 0.29 |
|  | Region: South vs. East | -0.344 | 0.159 | -.075 | .03 | -0.66 ; -0.03 |
|  | Region: West vs. East | -0.163 | 0.153 | -.038 | .29 | -0.46 ; 0.14 |
|  | Region: North vs. East | -0.134 | 0.133 | -.039 | .31 | -0.4 ; 0.13 |
|  | Type of insurance: Private vs. Statutory | 0.040 | 0.118 | .010 | .73 | -0.19 ; 0.27 |
|  | Digital literacy | 0.021 | 0.032 | .022 | .50 | -0.04 ; 0.08 |
|  | Performance Expectancy | 0.434 | 0.048 | .410 | . <.001 | 0.34 ; 0.53 |
|  | Effort Expectancy | 0.366 | 0.073 | .236 | . <.001 | 0.22 ; 0.51 |
|  | Social Influence | 0.186 | 0.036 | .173 | <.001 | 0.11 ; 0.26 |
|  | Facilitating Condition | 0.127 | 0.063 | .085 | .045 | 0 ; 0.25 |
|  | Perceived Privacy and Security | 0.121 | 0.040 | .104 | .003 | 0.04 ; 0.2 |

**Note:** Table displays the regression coefficients for our regression model. SE = standard error for regression coefficient B; 95% CI presents upper and lower bound for the 95% confidence interval for B.
